# RNA binding protein IGF2BP1 synergizes with ETV6-RUNX1 to drive oncogenic signaling in B-cell Acute Lymphoblastic Leukemia

**DOI:** 10.1186/s13046-023-02810-1

**Published:** 2023-09-05

**Authors:** Gunjan Sharma, Tiffany M. Tran, Ishu Bansal, Mohammad Sabique Beg, Ruchi Bhardwaj, Jaspal Bassi, Yuande Tan, Amit Kumar Jaiswal, Christine Tso, Ayushi Jain, Jay Singh, Parthaprasad Chattopadhyay, Archna Singh, Anita Chopra, Sameer Bakhshi, David Casero, Dinesh S. Rao, Jayanth Kumar Palanichamy

**Affiliations:** 1 Department of Biochemistry, All India Institute of Medical Sciences, Room 4008, Convergence Block, New Delhi, 110029 India; 2 Department of Pathology and Laboratory Medicine, David Geffen School of Medicine, University of California, Los Angeles, California USA; 3 F. Widjaja Foundation Inflammatory Bowel and Immunobiology Research Institute, Cedars-Sinai Medical Center, Los Angeles, California USA; 4 Department of Laboratory Oncology, Dr B.R Ambedkar Institute Rotary Cancer Hospital, All India Institute of Medical Sciences, New Delhi, India; 5 Department of Medical Oncology, Dr B.R Ambedkar Institute Rotary Cancer Hospital, All India Institute of Medical Sciences, New Delhi, India

**Keywords:** RNA binding protein, Leukemia, *ETV6::RUNX1* translocation, B-ALL, NFκB, PI3K pathways

## Abstract

**Background:**

Acute lymphoblastic leukemia (ALL) is the most common pediatric hematological malignancy, with *ETV6::RUNX1* being the most prevalent translocation whose exact pathogenesis remains unclear. IGF2BP1 (Insulin-like Growth Factor 2 Binding Protein 1) is an oncofetal RNA binding protein seen to be specifically overexpressed in *ETV6::RUNX1* positive B-ALL. In this study, we have studied the mechanistic role of IGF2BP1 in leukemogenesis and its synergism with the ETV6::RUNX1 fusion protein.

**Methods:**

Gene expression was analyzed from patient bone marrow RNA using Real Time RT-qPCR. Knockout cell lines were created using CRISPR-Cas9 based lentiviral vectors. RNA-Seq and RNA Immunoprecipitation sequencing (RIP-Seq) after IGF2BP1 pulldown were performed using the Illumina platform. Mouse experiments were done by retroviral overexpression of donor HSCs followed by lethal irradiation of recipients using a bone marrow transplant model.

**Results:**

We observed specific overexpression of IGF2BP1 in *ETV6::RUNX1* positive patients in an Indian cohort of pediatric ALL (*n*=167) with a positive correlation with prednisolone resistance.

IGF2BP1 expression was essential for tumor cell survival in multiple *ETV6::RUNX1* positive B-ALL cell lines. Integrated analysis of transcriptome sequencing after IGF2BP1 knockout and RIP-Seq after IGF2BP1 pulldown in Reh cell line revealed that IGF2BP1 targets encompass multiple pro-oncogenic signalling pathways including TNFα/NFκB and PI3K-Akt pathways. These pathways were also dysregulated in primary *ETV6::RUNX1* positive B-ALL patient samples from our center as well as in public B-ALL patient datasets. IGF2BP1 showed binding and stabilization of the *ETV6::RUNX1* fusion transcript itself. This positive feedback loop led to constitutive dysregulation of several oncogenic pathways.

Enforced co-expression of ETV6::RUNX1 and IGF2BP1 in mouse bone marrow resulted in marrow hypercellularity which was characterized by multi-lineage progenitor expansion and strong Ki67 positivity. This pre-leukemic phenotype confirmed their synergism *in-vivo*. Clonal expansion of cells overexpressing both ETV6::RUNX1 and IGF2BP1 was clearly observed. These mice also developed splenomegaly indicating extramedullary hematopoiesis.

**Conclusion:**

Our data suggest a combined impact of the ETV6::RUNX1 fusion protein and RNA binding protein, IGF2BP1 in activating multiple oncogenic pathways in B-ALL which makes IGF2BP1 and these pathways as attractive therapeutic targets and biomarkers.

**Supplementary Information:**

The online version contains supplementary material available at 10.1186/s13046-023-02810-1.

## Background

Acute lymphoblastic leukemia (ALL) is the most common pediatric malignancy with ~85% being of B-cell origin (B-ALL) [1]. B-ALL is characterized by the presence of different translocations including *BCR::ABL, ETV6::RUNX1, TCF3::PBX1, KMT2A* fusion rearrangement, and several novel subtypes classified by gene expression signatures [2]. *ETV6::RUNX1* is the most common translocation with an incidence of ~25% in Western countries and a lower incidence in India and its neighbouring countries [3, 4]. The *ETV6::RUNX1* fusion occurs as an early, prenatal event in-utero and results in the formation of a pre-leukemic clone [5, 6] which converts to ALL at a later stage after acquiring secondary mutations [7, 8]. Despite its association with a good prognosis, some *ETV6::RUNX1* patients have an overall poorer prognosis, usually associated with relapse [9].

*ETV6::RUNX1* is a weak oncogene and its enforced expression in mouse hematopoietic stem cells (HSCs)/committed progenitors does not result in the development of B-ALL. Leukemia development was observed at a low penetrance when combined with the loss of one of the alleles of *Kdm5c*/*Pax5*/*Cdkn2a* or when exposed to infections [10–12].

IGF2BP1, an oncofetal RNA binding protein (RBP), is known to be overexpressed in several cancers [13, 14] including B-ALL [15, 16]. IGF2BP1 has been seen to promote leukemic stem cell (LSC) maintenance and survival [17]. IGF2BP1 overexpression has been reported in various human cancers, including hepatocellular carcinomas, melanomas [18], neuroblastomas [19], breast cancer (overexpressed in ~58.5% of cases) [20], colorectal cancer (stage III/IV: 61.3% and stage I/II: 40%) [21], and ovarian cancer (69%) [22], and is also associated with poor prognosis and aggressive tumor behavior. A GWAS study identified IGF2BP1 expression to be unique in *ETV6::RUNX1* translocation positive B-ALL patients [16]. *ETV6::RUNX1* transcript is a known target of IGF2BP1 [15]. Overexpression of *IGF2BP1* in the bone marrow has been found to be highly specific for diagnosing the presence of the *ETV6::RUNX1* translocation [15].

In this study, we extend our current knowledge in understanding the role of IGF2BP1 overexpression in expediting the pathogenesis of *ETV6::RUNX1* positive B-ALL. We demonstrate *IGF2BP1* overexpression in *ETV6::RUNX1* B-ALL patient samples and also show the presence of prednisolone resistance and relapses in a subset of these patients. CRISPR mediated knockout of *IGF2BP1* or *ETV6::RUNX1* led to reduced tumor cell survival and reversal of prednisolone resistance. The transcriptome regulated by IGF2BP1 included numerous pro-oncogenic signalling pathways including the TNFα/NFκB and the PI3K-AKT pathways. We validated the upregulation of some of these putative IGF2BP1 target genes in a B-ALL patient cohort.

A bone marrow transplant model co-expressing ETV6::RUNX1 and IGF2BP1 in lethally irradiated mice led to the development of a pre-leukemic phenotype that included clonal expansion in the bone marrow which was primarily led by uncommitted, progenitor proliferation. Overall, our results suggest that IGF2BP1 plays an important role in the pathogenesis of *ETV6::RUNX1* leukemia through multiple oncogenic pathways and can be utilized as an ideal therapeutic target for this particular subtype.

## Methods

### Patient sample collection and processing

Treatment naïve B-ALL patient bone marrow (BM) samples were collected from March 2016 to December 2020 at BR Ambedkar Institute Rotary Cancer Hospital at AIIMS, New Delhi. The study was approved by the Institutional Ethics and Biosafety Committees (IEC-1950/1.04.2016, RP-20/2016). Samples were collected after informed consent from a guardian and assent was taken from children >7 years of age in accordance with the Declaration of Helsinki regulations. In addition, some archival BM samples, preserved from previous studies, were also utilized after proper ethics clearance. Peripheral blood from healthy controls was also collected.

### Cell culture

The human cell lines HEK 293T, Reh, RS4;11, THP1, RL, Jurkat, NALM6 and the murine pre-B-ALL cell line 7OZ/3 were obtained from American Type Culture Collection (ATCC) and maintained as described previously [23]. AT-1, AT-2 and UoCB6 were kind gifts from Dr Russell Ryan from the University of Michigan and have been verified by STR analysis.

### RNA isolation and real-time PCR

Total RNA was extracted from BM/cell lines using the TRIzol (Takara) method. 500-1000ng of RNA was reverse transcribed to cDNA using MMLV RT (Thermo Scientific, USA). Real Time PCR was performed to quantify gene expression using TB Green Premix Ex II (Takara)*. RNA Polymerase II, PPIA* and *HGPRT* were used as internal controls for mammalian cell lines and m*Gapdh* and m*L32* for murine cell lines*.* ΔΔCt method was used to compare the gene expression [24].

### CRISPR/Cas9 KO

Reh-Cas9 was created by transducing Reh with LentiCas9-GFP overexpressing lentiviruses. Guide RNAs were designed using the Zhang lab website (http://crispr.mit.edu/) and cloned into pLKO5-EFS-tRFP vector [25]. Lentiviruses and retroviruses were generated as previously described [26, 27].

### Statistical analysis

The patients were followed up in the Medical Oncology department, BRAIRCH, AIIMS. The last follow up was upto December 31, 2021. Overall survival (OS) was calculated as the time duration from the date of diagnosis to death or last follow-up. Event-free survival (EFS) was calculated as the time duration from the date of diagnosis to the date of the last follow-up or the first event (relapse or death). The probability of EFS and OS was calculated by the Kaplan-Meier method, with the differences compared using a two-sided log-rank test. All *in-vitro* experiments were repeated at least thrice. Mouse experiments were done at least twice. Comparison between different groups was done using Mann Whitney (two groups) /Kruskal-Wallis (more than two groups) statistical tests wherever applicable using GraphPad Prism software version 5 or SPSS statistical software package v20. A *p*-value of *<0.05* was considered to be significant. Additional methods and reagent details are provided in Supplementary Methods and Tables 1-5.

### Data availability statement

The analyzed RNA-Seq and RIP-Seq data can be found in a data supplement available with the online version of this article. Raw reads are available in the SRA database with BioProject ID PRJNA837729.

We have utilized some public ALL transcriptomic datasets to corroborate our data from the cBioportal (https://www.cbioportal.org/). The results published here are in whole or part based upon data generated by the Therapeutically Applicable Research to Generate Effective Treatments (https://ocg.cancer.gov/programs/target) initiative, phs000463 and phs000464. The data used for this analysis are available at ‘https://portal.gdc.cancer.gov/projects’.

## Results

### *IGF2BP1* expression is specific to *ETV6::RUNX1* positive patient samples

**Fig. 1:**
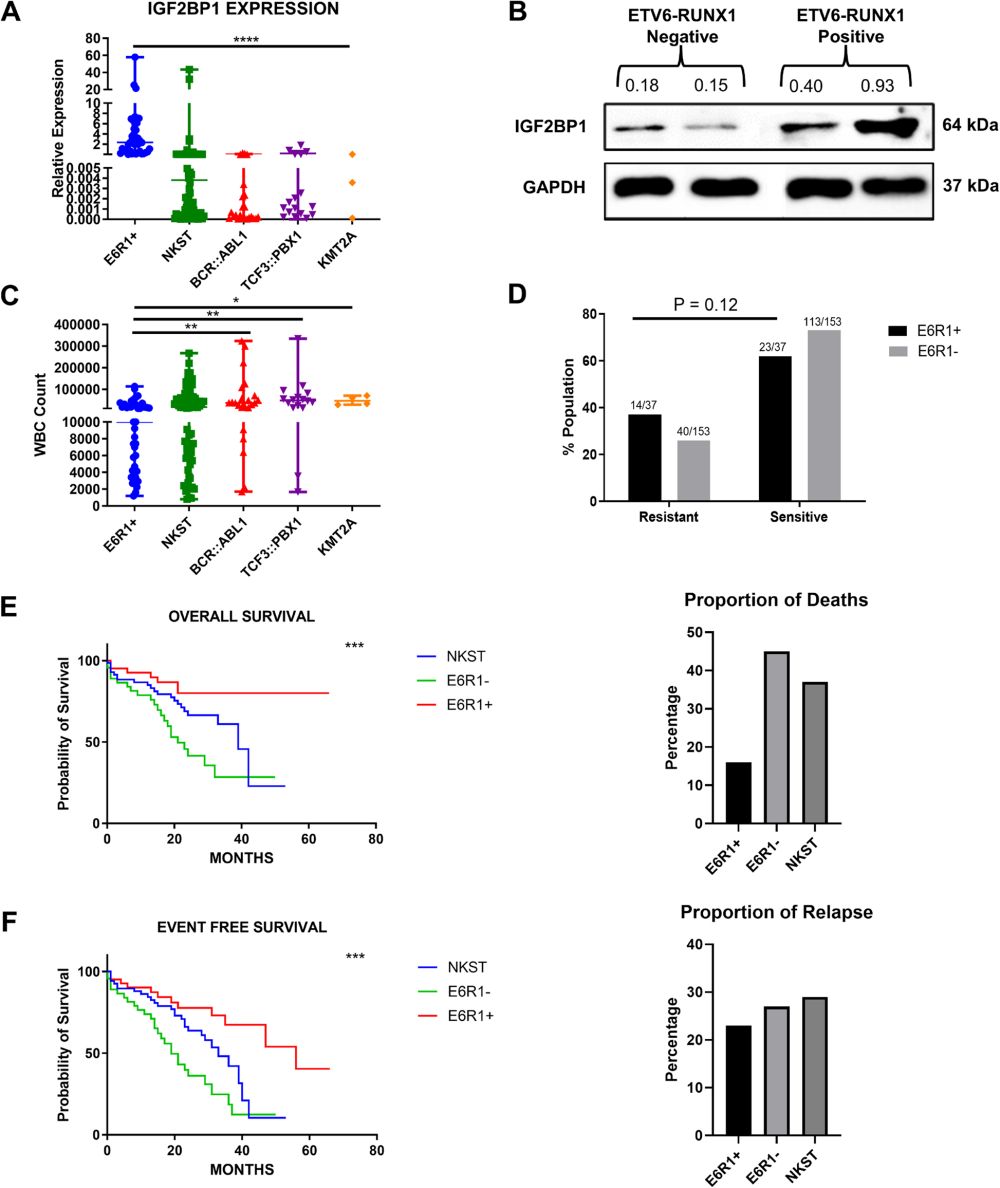
Expression of *IGF2BP1* and correlation with patient prognostic parameters. **A**) Real time expression data showing *IGF2BP1* overexpression in *ETV6::RUNX1* translocation positive patients (*n*=43), patients with no known sentinel translocation (*n*=76, includes patients with altered cytogenetics, *n*=13) and patients with other translocations (*n*=48, *BCR::ABL1* (*n*=27)*, TCF3::PBX1* (*n*=17)*, KMT2A* (*n*=4)*)* **B**) Western Blot showing protein expression of IGF2BP1 in *ETV6-RUNX1* translocation positive and negative samples. GAPDH: internal control. **C)** WBC counts in B-ALL patient groups (****p<0.0001, ***p < 0.001, **p < 0.01, *p < 0.05) **D**) Percentage of prednisolone resistant and sensitive patients in *ETV6::RUNX1* positive and negative subgroups (chi-square-test) **E**) Overall and **F**) Event free survival of B-ALL patients (*N* = 163) belonging to *ETV6::RUNX1* translocation positive group (E6R1+) (*n* = 43 ), other translocations group (E6R1-) (*n* = 44 ), No known sentinel translocation group (NKST) (*n* =76) (Kaplan-Meier method with Log-rank test; ***p < 0.001)

We had earlier reported the overexpression of *IGF2BP1* by RT-qPCR in the bone marrow of patients belonging to the *ETV6::RUNX1* subtype in a cohort of Indian patients (*n*=114) [28]. Additional samples were added to the same cohort (Total *n*=167). Among the 43 *ETV6-RUNX1* positive patients, 95.3% (41/43) showed IGF2BP1 overexpression (relative expression cutoff > 0.1) (Fig. 1A). Immunoblotting of protein lysates from two *ETV6-RUNX1* positive patients confirmed overexpression of IGF2BP1 protein compared to *ETV6-RUNX1* negative patients (Fig. 1B).

The presence of the *ETV6::RUNX1* translocation also correlated with lower White Blood Corpuscle (WBC) counts (Fig. 1C). For analyzing prednisolone response, the samples were stratified into *ETV6::RUNX1* positive and negative groups. The negative group included patients from the other translocations group (*BCR::ABL1*, *TCF3::PBX1* and *KMT2A*) and the No Known Sentinel Translocation (NKST) group. We observed a higher proportion of Prednisolone Poor Responders (PPRs) among the *ETV6::RUNX1* positive patients (38% vs 26%) which was statistically non-significant (Fig. 1D).

The presence of this translocation correlated with a better overall survival (OS) as well as a lower percentage of deaths (*p*=0.034, NKST vs E6R1+ and *p*<0.0001, E6R1- vs E6R1+) (median survival 39 months for NKST and 21 months for E6R1- patients and >60 months for E6R1+ patients) (Fig. 1E) which corroborates with existing literature [29]. Despite showing a better event-free survival (EFS) (*p*=0.017, NKST vs E6R1+ and *p*<0.0001, E6R1- vs E6R1+) (median survival was 56 months for E6R1+ patients compared to 33 months for NKST and 19 months for E6R1- patients), around 23% of the *ETV6::RUNX1* positive patients relapsed (Fig. 1F).

*IGF2BP1* expression was specific to *ETV6::RUNX1* positive patient samples. Despite having a better OS and EFS, this subgroup had a higher incidence of prednisolone resistance.

### IGF2BP1 loss of function inhibits B-ALL cell proliferation and reverses prednisolone resistance

**Fig. 2:**
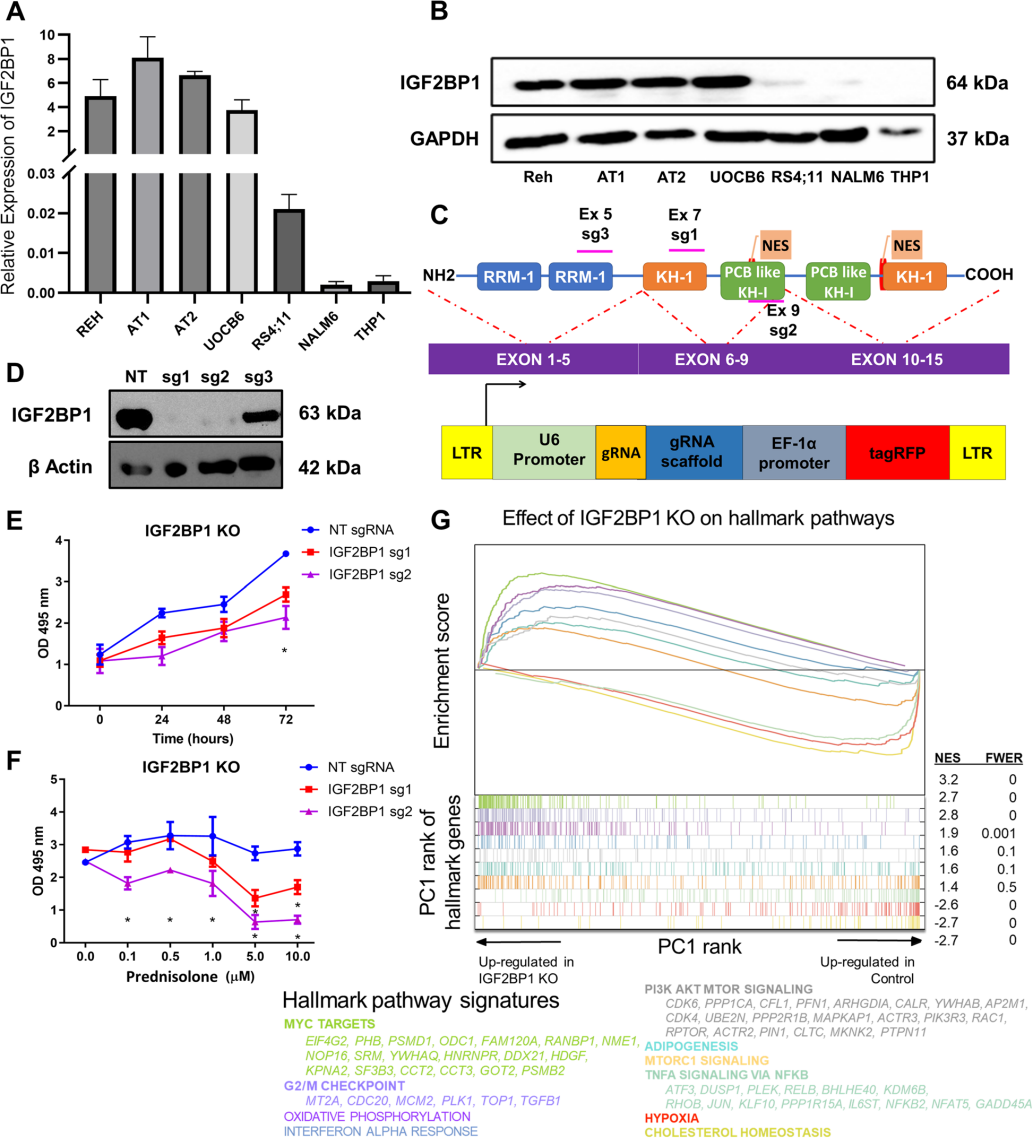
Effect of *IGF2BP1* knockout in *ETV6::RUNX1* positive Reh cell line **A**) mRNA expression of *IGF2BP1* in different leukemia cell lines (Reh, AT1, AT2, UoCB6 – *ETV6-RUNX1* translocation positive cell lines and RS4;11, NALM6, THP1 – *ETV6-RUNX1* translocation negative cell lines) analyzed by qRT-PCR. *POLR2A* and *HGPRT* were used as internal controls. **B**) Western Blot showing protein expression of IGF2BP1 in multiple cell lines. GAPDH was used as the internal control. **C**) Schematic for cloning of guide RNAs targeted against different exons of *IGF2BP1* in pLKO5-tRFP vector **D**) Western blotting after knockout of IGF2BP1 in Reh Cas9-GFP cells with β-Actin as the loading control; NT (Non-targeting control) **E**) Cell proliferation of different IGF2BP1 KO clones as determined by MTS assay (t-test; *p < 0.05, **p < 0.01, ***p < 0.001, ****p < 0.0001) **F**) Effect of Prednisolone induced cytotoxicity in Reh-Cas9 cells after *IGF2BP1* KO using MTS assay (IC50 ~ 1 μM for sg1/2) G) Gene Set Enrichment Analysis (GSEA): Hallmark pathways enriched after RNA-Seq of IGF2BP1 KO in Reh-Cas9 cells; The x axis represents the pre-ranked list of genes based on PC1 loadings, which segregates between genes more expressed in IGF2BP1 KO cells (left) and wild type cells (right). Segment plots (bottom) highlight the position of genes from hallmark pathways in the pre-ranked list. The vertical axis in line plots (top) represents the cumulative Enrichment Score (ES) from GSEA, and NES is the overall normalized enrichment score (with FWER=familywise error rate) for each selected pathway. Color-coded names for some genes in selected pathways are shown

*IGF2BP1* expression was specific to the *ETV6::RUNX1* translocated cell lines (Reh, UoCB6, AT1, and AT2) when compared to the leukemic cell lines lacking the *ETV6-RUNX1* translocation (NALM6, RS4;11, and THP1), both at the mRNA and protein levels, (Fig. 2A-B, Supplementary figure S1). This corroborated with the *IGF2BP1* expression data from the patient samples.

To examine the effects of the loss of IGF2BP1, knockout was performed in Reh-Cas9 cell line using 3 different sgRNAs (sg1-3) which were designed and cloned as described previously [30] (Fig. 2C). A Western blot for IGF2BP1 revealed that sg1 and sg2 caused complete knockout of IGF2BP1 protein, whereas sg3 caused partial knockout (Fig. 2D). The guides sg1 and sg2 were selected for further phenotypic characterization and analysis.

Complete knockout of *IGF2BP1* led to a significant decrease in cell proliferation as assessed by the MTS assay. After 72 hours of transduction, the cell viability was lower by ~26% for sg1 and ~41% for sg2, relative to the non-targeting gRNA (NT) (Fig. 2E). *IGF2BP1* knockout reversed prednisolone resistance and cells showed sensitivity to prednisolone with IC50 ~1 μM for both sg1 and sg2 (Fig. 2F). Cells with the non-targeting gRNA (NT) were viable even at a 10 μM concentration of prednisolone, confirming resistance [31]. There was a drastic fall in cell viability after IGF2BP1 KO by sg1 (~60%) and sg2 (~75%) after treatment with 5 μM prednisolone.

IGF2BP1 is specifically expressed in ETV6::RUNX1 positive B-ALL cell lines and its knockout reduces tumor cell proliferation and prednisolone resistance in Reh cell line.

### Integrated transcriptome and immunoprecipitation sequencing reveals overlap between IGF2BP1 and ETV6::RUNX1 regulated oncogenic pathways

To begin to unravel the mechanistic basis of our observations, the gene expression profile of *IGF2BP1* KO Reh cells was analysed by RNA-Seq. 88 transcripts showed upregulation and 270 transcripts showed downregulation (at least 2-fold change, *p*<0.05) after IGF2BP1 knockout. Gene Set Enrichment Analysis (GSEA) [32] revealed a significant negative enrichment of the TNFα signalling via NFκB, hypoxia and cholesterol metabolism pathways in KO cells as compared to non-targeting controls. A significant positive enrichment was found for pathways regulating cell cycle and proliferation including G2M checkpoint and the MYC targets (Fig. 2G).

**Fig. 3:**
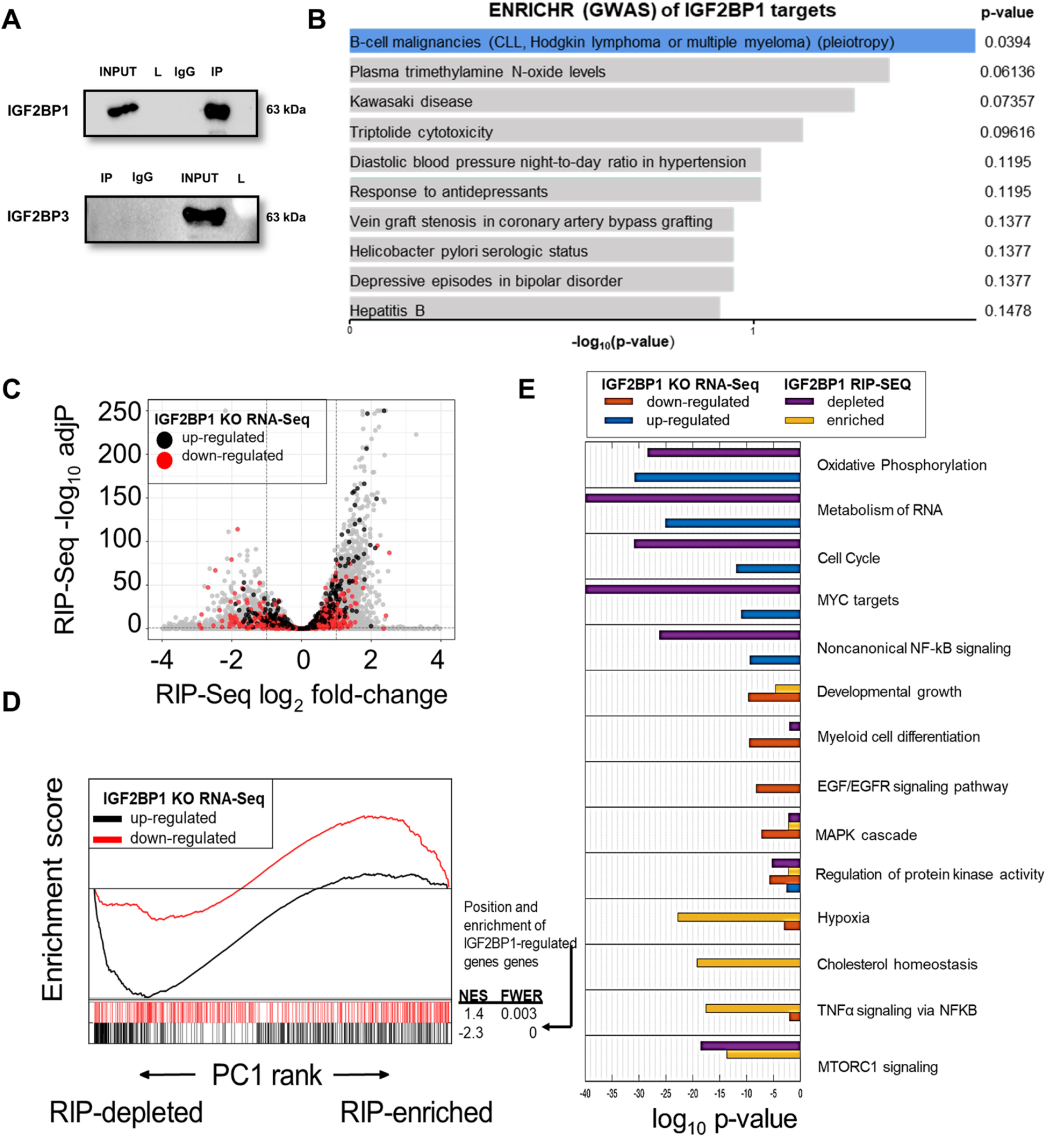
Identification of downstream pathways of IGF2BP1 in B-ALL. **A**) Western Blotting after RNA Immunoprecipitation of IGF2BP1 in Reh cell line; Specificity of the immunoprecipitation established by Western blotting analysis for another family member, IGF2BP3. Input is the precleared Reh cell lysate; L: Ladder; IgG: Mouse IgG; IP: IGF2BP1 Pulldown **B**) Output of EnrichR package used to categorize the IGF2BP1 RIP targets using GWAS catalogue **C**) Volcano plot of IGF2BP1 RIP-Seq data in Reh cell line. Genes up and down-regulated (RNA-Seq) after IGF2BP1 KO are highlighted as black and red dots respectively **D**) GSEA analyzing the association between enrichment in IGF2BP1 RIP-Seq samples and genes regulated in IGF2BP1 KO cells. X axis represents the pre-ranked list of genes based on RIP-Seq PC1 loadings, which segregates between genes enriched (right) and depleted (left) in IP samples. Segment plots (bottom) highlight the position of genes strongly up/down regulated in IGF2BP1 KO cells. The vertical axis in line plots (top) represents the cumulative Enrichment Score (ES) from GSEA, and NES is the overall normalized enrichment score (with FWER=familywise error rate) for each gene set **E**) Pathway enrichment results for several gene classes: genes strongly up/down regulated in *IGF2BP1* KO cells and genes strongly enriched or depleted in the IGF2BP1 RIP-Seq dataset. For each pathway, shown are the hypergeometric adjusted *p*-values for each gene class

To gain insights into the mRNA interactome of IGF2BP1, we performed RNA Immunoprecipitation followed by high throughput sequencing (RIP-Seq) (Fig. 3A). Application of SETEN [33], a GSEA-based tool for RNA-binding proteins, revealed enrichment of putative IGF2BP1 targets in numerous pro-oncogenic signaling pathways. The ENRICHR tool [34] classified IGF2BP1 target transcripts to be involved in B-cell and myeloid malignancies using the GWAS catalogue database. GO biological processes and molecular functions revealed enrichment for RNA processing, translation and RNA degradation pathways (Fig. 3B, Supplementary Figure S2A-D).

An integrated analysis of the IGF2BP1-KO RNA-Seq and IGF2BP1 RIP-Seq in Reh cells was performed. Initially, we observed that IGF2BP1 putative targets (RIP-Seq log2 fold change>0) comprised both up and down-regulated genes after IGF2BP1 deletion (Fig. 3C). However, integration of both datasets using GSEA (Fig. 3D) showed that only the set of genes most downregulated after IGF2BP1 deletion were significantly classified as IGF2BP1 putative targets (highest RIP-Seq enrichment, familywise error-rate *p*-value<0.003, Fig. 3D). This suggests that IGF2BP1 preferentially promotes RNA stability in Reh cells. Interestingly, genes up-regulated after IGF2BP1 KO were preferentially not enriched in the RIP data (familywise error-rate *p*-value<0.001, Fig. 3D) suggesting that these genes are indirectly regulated by IGF2BP1 (Fig. 3C-D)

**Fig. 4:**
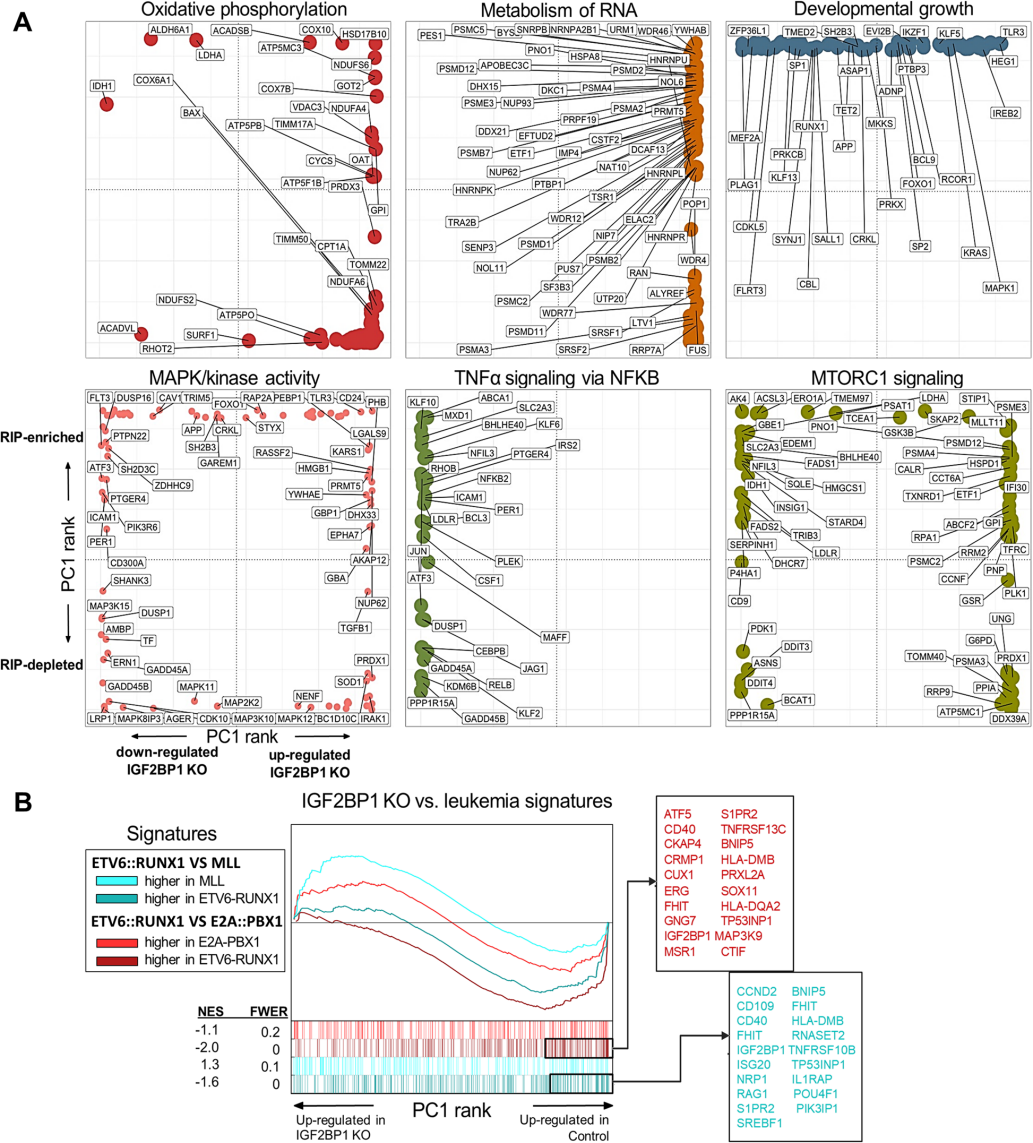
Functional enrichment analysis of IGF2BP1 regulated pathways in B-ALL. **A**) Rank distribution plots of genes in selected functional categories. For each pathway, the x-axis represents the gene’s rank based on RNA-Seq PC1 loadings, which segregates between genes more expressed in IGF2BP1 KO cells (right) and wild type cells (left). The y axis represents the gene’s rank based on RIP-Seq PC1 loadings, which positions RIP-enriched genes on top, and depleted genes at the bottom. Shown are selected gene names of top-ranked genes in each pathway **B**) GSEA results of leukemia-associated genes in IGF2BP1 KO Reh cells. X axis represents the pre-ranked list of genes based on PC1 loadings, which segregates between genes more expressed in IGF2BP1 KO cells (left) and wild type cells (right). Segment plots (bottom) highlight the position of genes from several genesets identified as up/down regulated in two independent studies comparing *ETV6::RUNX1* with *KMT2A* and *TCF3::PBX1* rearranged leukemias. The vertical axis in line plots (top) represents the cumulative Enrichment Score (ES) from GSEA, and NES is the overall normalized enrichment score (with FWER=familywise error rate) for each gene set. Color-coded names for some genes in selected gene sets are shown

Combined pathway analysis of IGF2BP1 KO/RIP gene sets revealed that some of the significantly dysregulated pathways after the knockout also had many genes enriched in the RIP-Seq suggesting direct binding and regulation (Fig. 3E, Supplementary Data). Examples include the TNFα/NFκB signaling and Hypoxia pathways. Oxidative phosphorylation was upregulated after knockout while cholesterol metabolism was downregulated. However, both were not RIP targets implying an indirect or downstream effect of *IGF2BP1* knockout on those pathways. A deeper analysis of genes within these pathways showed that some of the genes in the metabolism of RNA, oxidative phosphorylation, MTORC1 and MAPK pathways showed enrichment in the RIP dataset (Fig. 4A).

We then aimed to map IGF2BP1-induced gene expression changes to expression signatures of different patient subtypes. We applied GSEA to compare our *IGF2BP1*-KO data to differential gene expression between *ETV6::RUNX1*, *TCF3::PBX1* and *KMT2A* positive patients (from GSE65647, *n*=44) [23]. We also analyzed a dataset which compared *ETV6::RUNX1* positive patients’ gene expression (*n*=4) with normal CD19 positive B-cells (*n*=2) [35]. There was a significant overlap between genes overexpressed and pathways enriched in *ETV6::RUNX1* positive tumors from both the datasets and genes/pathways downregulated after deletion of *IGF2BP1*. For example, the TNFα/NFκB signaling and inflammatory signaling pathways were upregulated in the *ETV6::RUNX1* positive tumors while MYC targets and oxidative phosphorylation pathways were suppressed. The converse was seen after IGF2BP1 KO (Fig. 4B, Supplementary Figure S3-S5)

Taken together, these results suggest that IGF2BP1 binds to and regulates the expression of multiple pro-oncogenic signaling pathways in *ETV6::RUNX1* positive B-ALL cells. These pathways were also specifically enriched in the transcriptome of *ETV6::RUNX1* positive patients indicating a critical role for IGF2BP1 in this leukemia subtype.

### TNFα induced NFκB and PI3K-Akt signalling pathways are activated in *ETV6::RUNX1* positive B-ALL by IGF2BP1

**Fig. 5:**
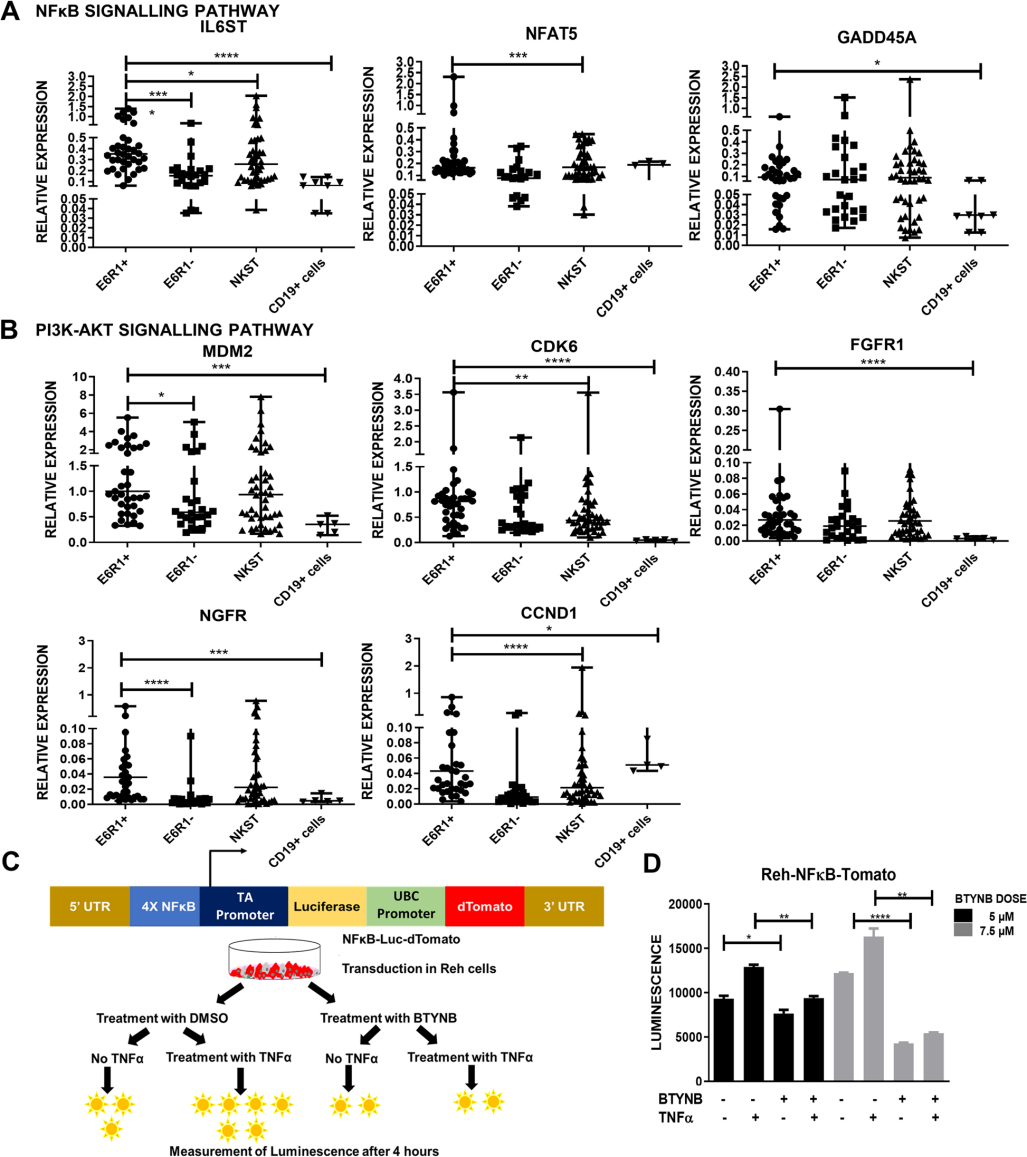
Functional validation of NFκB and PI3K pathways: Real Time PCR-based validation of IGF2BP1 targets identified from the RIP-Seq data belonging to the **A**) NFκB and **B**) PI3K-Akt pathways in the Indian patient cohort (Total *n* = 111; E6R1+ *n*= 39, E6R1- (Other translocations) *n*= 26, No translocation *n*= 46) and MACS sorted CD19+ B-cell population as controls (*n*=5) (t test; *p* * <0.05, ** <0.01, *** <0.005, **** <0.0001) **C**) Schematic of the NFκB-luciferase reporter assay **D**) NFκB induction by TNFα (25ng/mL) determined by measuring luciferase activity after BTYNB mediated inhibition of IGF2BP1 in Reh-NFκB-Luc-dTomato cells

We further validated genes from the TNFα/NFκB and PI3K-Akt pathways in our patient samples along with MACS enriched CD19+ B-cells as controls (Fig. 5A-B). Many of these genes including *IL6ST*, *MDM2, CDK6* and *NGFR* showed significant upregulation in the *ETV6::RUNX1* positive patients.

In order to validate and corroborate the pathway genes identified in our dataset, we analyzed the public ALL TARGET dataset which contains transcriptomic data from B-ALL patients (*n*=203). This data confirmed the overexpression of most of the genes in the TNFα induced NFκB and PI3K-Akt signalling pathways in the *ETV6::RUNX1* positive patients. There was a significant positive, linear correlation between *IGF2BP1* expression with some of its putative targets (*CDK6, MDM2, PRKCB, RAG1)* (Supplementary Figure S6*).* BCL2 is one of the major genes which drives glucocorticoid resistance in B-ALL by modulating the glucocorticoid receptor (GR: NR3C1) activity. *BCL2* was one of the targets of IGF2BP1 in the RIP and its levels were significantly downregulated after *IGF2BP1* knockout. The TARGET dataset also identified a significant positive correlation between *IGF2BP1* and *BCL2* expression. A previous study of IGF2BP1 knockdown in 697 cell line had identified that various stemness genes including *HOXB4*, *ALDH1A1*, *ALDH1A3* and the non-coding RNA *H19* were direct targets bound and stabilized by IGF2BP1 [17]. However, in Reh cell line, there was negligible expression of all these genes except *MYB*. *MYB* was found to be bound by IGF2BP1 and its expression was significantly downregulated after knockout. Their expression correlated significantly in the patient data from the TARGET dataset*)* (Supplementary Figure S7*)*. This points towards a unique transcriptomic program regulated by IGF2BP1 in *ETV6::RUNX1* positive cells.

To validate the regulatory role of IGF2BP1 in the non-canonical TNFα/NFκB pathway, we utilized BTYNB, a functional inhibitor of IGF2BP1 [36]. In *ETV6::RUNX1* positive cell lines (Reh, AT1, AT2, and UOCB6), treatment with BTYNB (2 to 15 μM) resulted in a dose-dependent decrease in cell proliferation as measured after 72 hours (Supplementary Figure S8*)*. This was accompanied by a reduction in the expression of both *IGF2BP1* and the *ETV6::RUNX1* fusion transcript, as measured by qPCR (Supplementary Figure S9). However, BTYNB had no effect on the proliferation of RL and Jurkat cell lines, which express minimal levels of IGF2BP1 (Supplementary Figure S8).

Reh cells were transduced with NFκB-Luc-dTomato plasmid [37] which consists of the NFκB consensus sequence upstream of the Luciferase reporter gene. These cells showed an increase in luminescence after treatment with TNFα. Pre-treatment of these cells with BTYNB led to a loss of this luminescence induction in a dose dependent manner (Fig. 5C-D).

The expression of some putative targets from the NFKB and PI3K-AKT signalling pathways were analyzed by qPCR and Western blotting. In *ETV6::RUNX1* positive cell lines, functional inhibition of IGF2BP1 using BTYNB significantly decreased the expression of these targets which was not observed in the *ETV6::RUNX1* negative cell lines (Supplementary Figures S10-12).

These results conclusively demonstrate that IGF2BP1 regulates the TNFα induced NFκB and PI3K-Akt signalling pathways in *ETV6::RUNX1* positive B-ALL and its inhibition leads to reduced tumor cell survival and reduced expression of these pathway targets.

### *ETV6::RUNX1* fusion transcript is stabilized by IGF2BP1

**Fig. 6:**
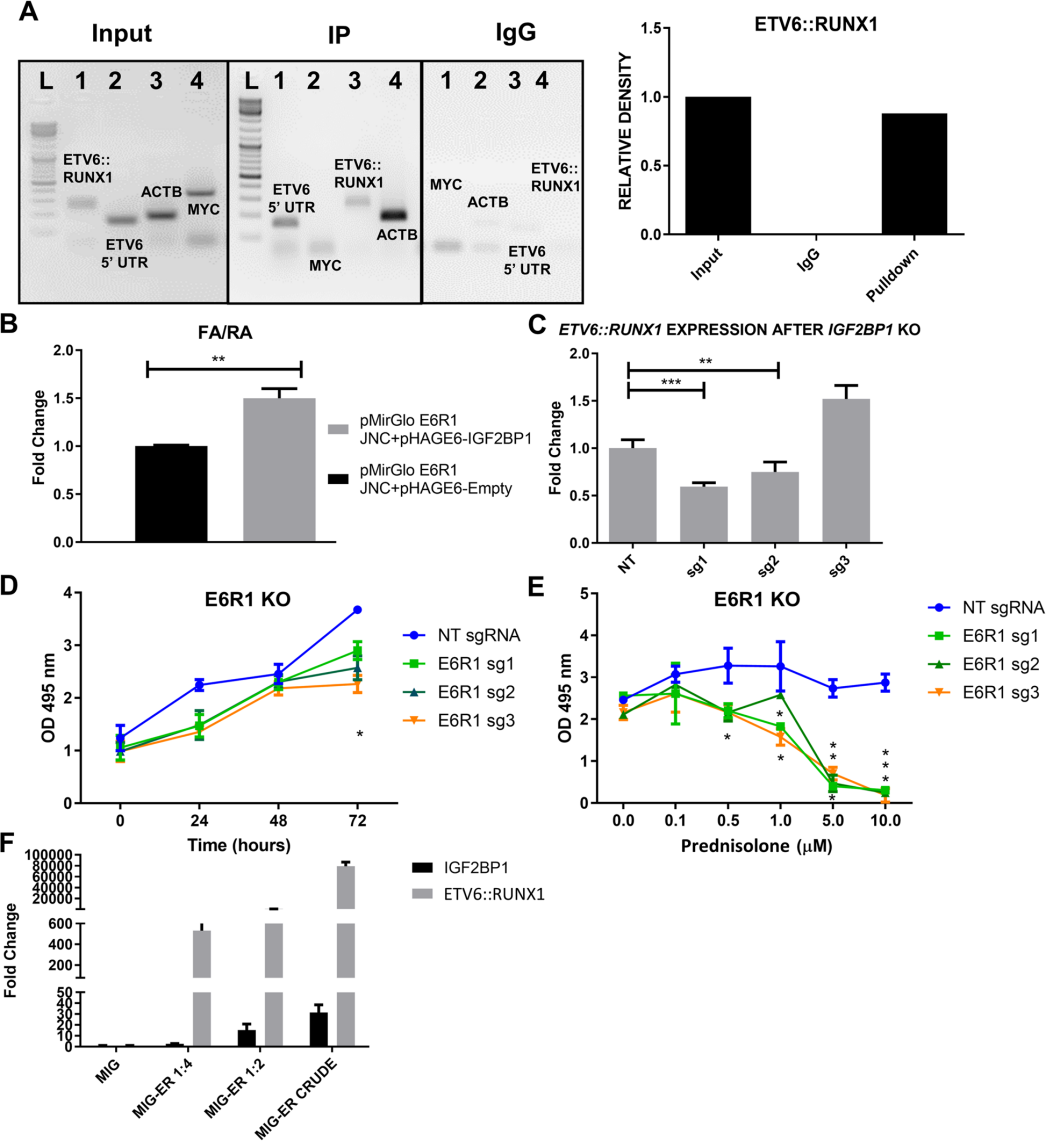
Positive feedback between ETV6::RUNX1 and IGF2BP1 **A**) RT-PCR of both Input and Immunoprecipitated RNA fraction for different known targets of IGF2BP1:* ETV6::RUNX1* junction (298 bp), MYC CRD region (357 bp) and 5’ UTR of ETV6 (154 bp) followed by agarose gel electrophoresis. This reveals ETV6::RUNX1 and 5’-ETV6 as targets; β-actin 3’ UTR (184 bp) was used as a positive control **B**) Dual luciferase assay in 293T cells reveals stabilization of the *ETV6::RUNX1* junction by IGF2BP1 **C**) Effect of *IGF2BP1* KO on *ETV6::RUNX1* fusion transcript stability as determined by qRT-PCR **D**) Cell proliferation assay (MTS) shows reduced proliferation of *ETV6::RUNX1* KO Reh-Cas9 cells **E**) Reversal of prednisolone resistance after *ETV6::RUNX1* KO in Reh-Cas9 cells determined after 72 hours using MTS assay (IC50=1.6/2/3.5 μM for sg1/2/3 respectively) (t-test; *p* * <0.05, ** <0.01, *** <0.005, **** <0.0001) F) Real Time PCR shows dose dependent increase in *IGF2BP1* levels after overexpression of *ETV6::RUNX1* fusion transcript in 7OZ/3

IGF2BP1-RIP followed by RT-PCR of immunoprecipitated RNA showed significant enrichment of the *ETV6::RUNX1* junction, 5’-UTR of *ETV6* and *ACTB (*positive control*)* in the RIP vs Input samples (Fig. 6A). In order to study the effect of IGF2BP1 on *ETV6::RUNX1* stability, we cloned the junction region downstream of firefly luciferase (Supplementary Figure S13A). Cotransfection of this vector with IGF2BP1 resulted in significantly increased luciferase activity (Fig. 6B). Complete knockout of *IGF2BP1 (sg1 and sg2)* significantly decreased *ETV6::RUNX1* transcript levels in Reh cells *(*Fig. 6C*)* which phenocopied the effect seen after IGF2BP1 inhibition by BTYNB (Supplementary Figure S9). This data demonstrated the regulation of IGF2BP1 by *ETV6::RUNX1* and its stabilization by the former.

We performed *ETV6::RUNX1* KO using guides targeting the *ETV6::RUNX1* junction or the 5’ ETV6 region as reported previously (Supplementary Figure S13B) [38] in Reh cell line. We observed that the KO cells showed a significant reduction in cell viability (21.13%, 30% and 38.33% lower than the NT cells for sg1, sg2 and sg3, respectively, after 72 hours of transduction) and an increased sensitivity to prednisolone. The cell viability started to decrease significantly even at 0.1 μM of prednisolone and reached 89.8%, 90.16% and 92.7% reduction for sgRNA1, sgRNA2 and sgRNA3, respectively at 10 μM of prednisolone, with IC50 values of 1.6, 2 and 3.5 μM for sg1, sg2 and sg3, respectively. This suggested the involvement of both the proteins in conferring a glucocorticoid-resistant phenotype to Reh cells (Fig. 6D-E).

We then proceeded to study the effect of *ETV6::RUNX1* overexpression on IGF2BP1 expression*. ETV6::RUNX1* fusion transcript has been shown to affect the functions of wild type *ETV6* as well as *RUNX1* in a dominant negative fashion [39]. We sub cloned the *ETV6::RUNX1* transcript from a pcDNA3.1-*ETV6::RUNX1* plasmid (a kind gift from Dr Anthony Ford, ICR, London) into a bicistronic, retroviral, murine overexpression vector (Supplementary Figure S13C). *ETV6::RUNX1* overexpressing retroviral particles were produced as described previously [40] and used to overexpress the same in a murine pre-B-ALL cell line, 7OZ/3 in three serial dilutions. There was a dose dependent increase in *Igf2bp1* levels (Fig. 6F).

Our data indicates that IGF2BP1 binds to and stabilizes the *ETV6::RUNX1* fusion transcript, which in turn upregulates IGF2BP1 expression, forming a positive feedback loop. Knockout of either IGF2BP1 or ETV6::RUNX1 reduces the cell proliferation and reverses the glucocorticoid resistance of Reh cells, indicating that both proteins are essential for the survival and growth of *ETV6::RUNX1* positive B-ALL cells.

### IGF2BP1 and ETV6::RUNX1 synergize to cause clonal progenitor expansion in the murine bone marrow

To directly assess the synergism between ETV6::RUNX1 and IGF2BP1, we undertook an *in-vivo* experiment to examine the effects of enforced expression of both transgenes. Murine bone marrow transplant experiments were performed using retroviruses, synthesized and used as previously described [40] (Supplementary Figure S14A). We cloned the human coding sequence of *IGF2BP1* into MIG (MSCV-IRES-GFP), a murine stem cell virus–based (MSCV) retroviral vector and used it along with the *ETV6::RUNX1* cloned in MICH (MSCV-IRES-mCherry). We confirmed the functionality of the vectors in expressing IGF2BP1 and ETV6::RUNX1 as well as the fluorescent markers (Supplementary Figure S14B–D and data not shown).

All groups had similar levels of engraftment as seen from the CD45.1 positivity (*data not shown)*. Clonal expansion was observed in the peripheral bleeds of mice expressing both ETV6::RUNX1 and IGF2BP1 (combination group) from weeks 4-16 with a constant increase. This was measured by the ratio of GFP+ mCherry+ cells to the total transfected cells (double positive ratio) (Supplementary Figure S15A-B).

Complete blood counts (CBC) at week 16 showed a decrease in mature B-cell counts after ETV6::RUNX1 overexpression and in the combination. There was an increase in total WBC counts after IGF2BP1 overexpression. In the combination, the number of mature red blood cells, platelets and neutrophils were significantly lower with an increase in immature reticulocyte counts (Supplementary Figure S16A-H).

**Fig. 7:**
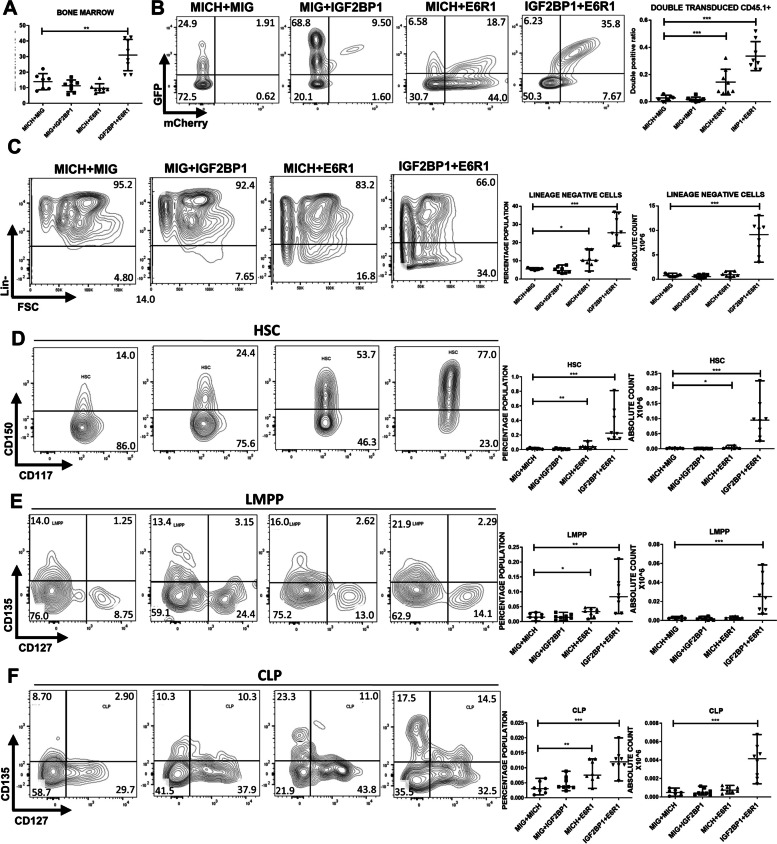
IGF2BP1 and ETV6::RUNX1 promote progenitor expansion in the mouse bone marrow **A**) Bone marrow cell counts showing significantly high numbers in the combination group expressing ETV6::RUNX1 and IGF2BP1 **B**) Double positive (GFP+ mCherry+) ratio in the bone marrow and representative FACS plots showing clonal expansion **C**)-**F**), Quantitation (percentage and absolute counts) of Lineage negative cells, Hematopoietic stem cells, lymphoid-primed multi-potential progenitors, common lymphoid progenitors in the bone marrow of the mice belonging to different groups at 16 weeks after bone marrow transplant with representative FACS plots (*n*= 8 in each group; t-test; *p* * <0.05, ** <0.01, *** <0.005, **** <0.0001)

To further characterize these hematopoietic changes, mice were euthanized after 16 weeks and hematopoietic organs collected for analysis. The bone marrow counts were significantly higher in the combination group with significant clonal expansion. Analysis of the progenitors revealed significant increase in the Lin- population, hematopoietic stem cells (HSCs), Lymphomyeloid Primed Progenitors (LMPP) and common lymphoid progenitors (CLPs) (percentages and absolute counts) in the combination group. The ETV6::RUNX1 group also showed a small but significant increase in progenitor output (Fig. 7). Hardy fraction analysis of the bone marrow in the combination showed an accumulation of the pre-B cell population implying a B-cell developmental block. (Supplementary Figure S17).

**Fig. 8:**
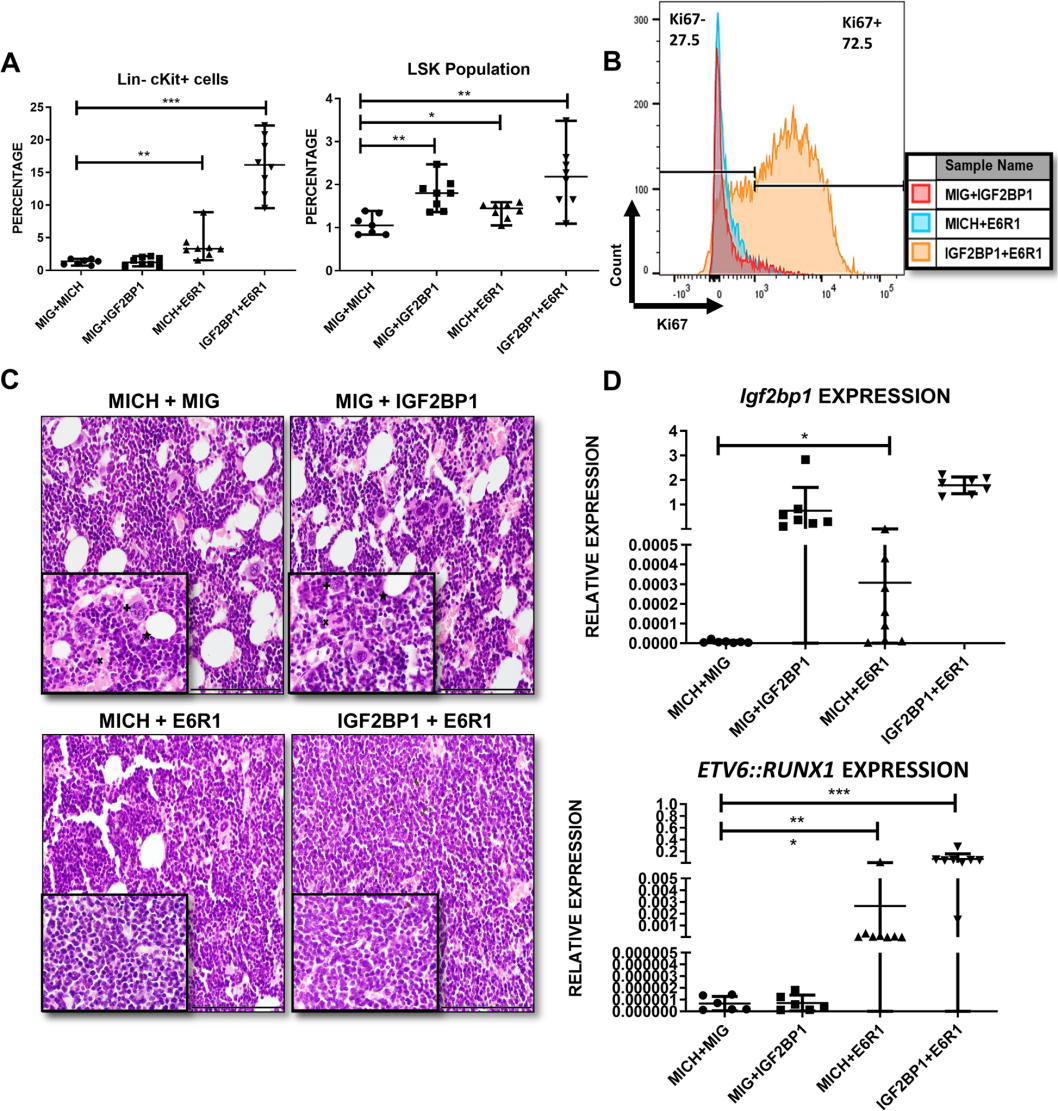
Histopathological analysis of bone marrow architecture after IGF2BP1 and ETV6::RUNX1 overexpression **A**) Quantitation of Lin- c-Kit+ cells and Lin- c-Kit+ Sca1+ cells (LSK population) in different mouse groups **B**) Ki67 positivity in the lineage negative cells of the bone marrow belonging to different groups **C**) Histologic imaging of bone marrow of mice belonging to different groups; *fat globules, +megakaryocytes, X: vascular space; Scale bar:100 microns (200X) **D**) Quantitation of *Igf2bp1* and ETV6::RUNX1 expression in mouse bone marrow (t-test; *p* * <0.05, ** <0.01, *** <0.005, **** <0.0001)

Previous studies have identified the Lin- c-Kit+ or the LSK (Lin- c-Kit+Sca1+) populations as leukemia initiating cells in different mouse models [41]. The bone marrow of the combination group showed a significant increase in both populations (Fig. 8A*)* with strong Ki67 positivity of the Lin- progenitor population implying an increased proliferation rate (Fig. 8B). The myeloid progenitors (common myeloid progenitors (CMP), granulocyte-monocyte progenitor (GMP) and megakaryocyte-erythroid progenitor (MEP)) were also significantly increased in the combination (Supplementary Figure S18).

The progenitor populations from the bone marrow were analyzed for fluorescent marker expression (double positive (DP): mCherry+ GFP+ and double negative (DN): mCherry-GFP-). In the combination group, there was an increase in the Lin- population in both the DN and DP fractions implying both a cell extrinsic and intrinsic mechanism for progenitor expansion. Interestingly, the HSCs and LMPPs were only increased in the DP fraction indicating a purely cell intrinsic mechanism (Supplementary Figure S19).

Histopathological examination of the bone marrow of mice expressing both ETV6::RUNX1 and IGF2BP1 showed marked hypercellularity along with a loss of normal architecture characterized by compressed vascular spaces, reduced fat globules and megakaryocytes (Fig. 8C). Spleens were significantly enlarged with clonal expansion (Supplementary Figure S20A-C). The spleen was populated by immature cells including HSCs implying extramedullary hematopoiesis (Supplementary Figure S20D). Histopathological analysis revealed loss of architecture with red pulp expansion and smaller germinal centers in the combination group (Supplementary Figure S20E).

RT-qPCR from the bone marrow of the mice showed a significant increase in endogenous *Igf2bp1* levels after ETV6::RUNX1 overexpression, validating our in-vitro finding. The levels of *ETV6::RUNX1* and *IGF2BP1* were highest in the combination group (Fig. 8D).

The mouse transplant experiments suggest that co-expression of ETV6::RUNX1 and IGF2BP1 in the murine bone marrow leads to clonal expansion of multiple progenitors including HSCs and the various B-lineage progenitors. This also leads to bone marrow hypercellularity and extramedullary hematopoiesis. Overexpression of ETV6::RUNX1 leads to an overexpression of endogenous Igf2bp1.

## Discussion

The molecular mechanism of ETV6::RUNX1 mediated leukemogenesis is incompletely understood. *ETV6::RUNX1* is known to be a weak oncogene unable to induce leukemia in the absence of secondary genetic alterations [12, 42, 43]. As detected from the bloodspots in newborns, this translocation shows a far higher prevalence, with very few going on to develop leukemia [44, 45] supporting the ‘two-hit’ model of its molecular pathogenesis [46]. *Cdkn2a* loss*,* alterations of *Epor, Ebf1, Jak1, Jak3, Il2rb, Stat5* and *Trp53* have all been shown to synergize with ETV6::RUNX1 to cause leukemia in mice [10, 42, 43, 47]. We had previously identified that the RBP IGF2BP1 was specifically overexpressed in the *ETV6::RUNX1* translocated B-ALL and validated the same in a larger cohort [28]. The importance of RBPs in the pathogenesis of various leukemias including the IGF2BP family is slowly being dissected [48]. Our findings add to this existing knowledge of dysregulated RBP expression in *ETV6::RUNX1* translocated B-ALL.

B-ALL treatment at our cancer center is done using the Indian Childhood Collaborative Leukemia Group (ICiCLe) Protocol [49]. One of the risk stratification parameters includes percentage of blasts on day 8 after treatment with high dose prednisolone which divides patients into good and poor responders (PGR/PPR) [50]. Glucocorticoid resistance has been linked to poor prognosis and decreased event free survival [51]. The presence of *ETV6::RUNX1* translocation appears to correlate with a trend of prednisolone resistance. Although, the presence of *ETV6::RUNX1* correlated with a good prognosis as previously reported [52], the event free survival was inferior in comparison to the overall survival. These findings highlight the varied nature of response to chemotherapy and poorer prognosis in at least a subset of the *ETV6::RUNX1* positive patients.

Previously, loss of function of IGF2BP1 in epithelial cell lines has been shown to decrease cell proliferation and cause apoptosis [14, 17]. We found that knockout of *IGF2BP1* led to reduced cell proliferation and reversal of prednisolone resistance in Reh, an *ETV6::RUNX1* positive cell line along with a downregulation of *BCL2* whose upregulation is usually associated with glucocorticoid resistance [53]. A similar phenotype was also observed after *ETV6::RUNX1* knockout. Interestingly, some of the pathways upregulated after *IGF2BP1* knockout are known to be upregulated after *ETV6::RUNX1* knockdown [54, 55]. Knockout of *IGF2BP1* also led to a decrease in the expression of *ETV6::RUNX1* transcript and overexpression of ETV6::RUNX1 lead to a dose dependent increase in *IGF2BP1* levels implying interdependency between the two genes. Previous studies have also identified ETV6::RUNX1 to be a target of IGF2BP1 and a 17q21 polymorphism within IGF2BP1 to be having a strong association with ETV6::RUNX1 positive B-ALL [16, 56].

A mechanistic examination of the transcriptome controlled by IGF2BP1 was analysed via a combined interrogation of the RIP-Seq/*IGF2BP1* KO RNA-Seq datasets which revealed enrichment of numerous pro-oncogenic pathways. Many pathways regulated by IGF2BP1 were also significantly associated with *ETV6::RUNX1* positive tumors, implying some degree of cooperativity between the two genes in disease pathogenesis.

IGF2BP1 appeared to bind and stabilize genes in the TNFα mediated non-canonical NFκB pathway which was also validated in our patient cohort. The non-canonical NFκB pathway is known to play an important role in inflammation [57]. In *ETV6::RUNX1* translocated tumors, it may be contributing to creating an inflammatory environment within the bone marrow niche for the maintenance or emergence of the leukemic clones as reported previously [58]. An in-vitro NFκB mediated luciferase reporter assay showed a decrease in luciferase activity after IGF2BP1 inhibition underscoring the clear role of IGF2BP1 in regulating this pathway.

Interestingly, the pathways which were negatively enriched in the *IGF2BP1* KO cells were all positively enriched in the *ETV6::RUNX1* positive tumors and vice versa. A recent study has demonstrated that ETV6::RUNX1 functions by competing for RUNX1 binding sites and leads to transcriptional repression of RUNX1 targets. The study also showed *ETV6::RUNX1* knockdown leading to an increase in the G2M checkpoint, E2F and MYC target pathways. These pathways were also upregulated after *IGF2BP1* knockout further establishing a role for their synergism [59].

RIP-Seq identified numerous pro-oncogenic pathways including the PI3K-Akt and MAPK pathways. Interestingly, some targets identified in RIP-Seq were not significantly downregulated after *IGF2BP1* knockout implying a multifactorial regulation of their mRNA stability. A qPCR validation of PI3K-Akt pathway genes (*MDM2*, *CDK6*, *CCND1*, *NGFR*) followed by a public dataset analysis demonstrated a significantly high expression in *ETV6::RUNX1* positive patients. ETV6::RUNX1 is known to increase MDM2 which normally degrades p53 [60]. Interestingly, MDM2 is also known to have a p53-independent role in childhood ALL where its elevated expression induces expression of p65 subunit of NFκB and augments chemoresistance [61]. We had previously demonstrated *CDK6* to be a target of IGF2BP3 in ALL suggesting some degree of overlap between targets of this family [23]. Cyclin D1 (CCND1) is required during the transition from G1 to S phase and is known to be overexpressed in B-cell lymphomas [62]. *CCND1* expression is associated with poor prognosis and relapse in childhood ALL [63]. High NGFR expression has been known to be associated with *ETV6::RUNX1* rearrangement [64].

To study the pathogenetic role of IGF2BP1 in leukemia development, we developed the first in-vivo model with enforced expression of IGF2BP1 in the hematopoietic system. The combination of IGF2BP1 and ETV6::RUNX1 led to a hypercellular bone marrow along with increased progenitor output across all lineages (HSCs, LMPPs, CLPs and myeloid progenitors). The peripheral blood showed a decrease in mature cells and increase in the immature cells over time. Together, these findings suggest that the combination of ETV6::RUNX1 and IGF2BP1 promotes skewing of BM development towards an immature phenotype. These progenitor cells were hyperproliferative as seen by Ki67 staining. There was significant clonal expansion of these double positive cells. ETV6::RUNX1 overexpression alone led to a small but significant increase in progenitor output as reported previously [10, 11, 47, 65]. This progenitor expansion appeared to be fuelled by both cell extrinsic and intrinsic mechanisms indicating that the combination led to an altered marrow niche favouring proliferation. IGF2BP1 has been shown to maintain stem cell properties by regulating *HOXB4*, *ALDH1A1* and *MYB* in leukemic cell lines [17]. However, most of these genes were negligibly expressed in Reh, implying that transcriptional program in *ETV6::RUNX1* positive cells is different than the *TCF3::PBX1* positive 697 cell line. Histopathological analysis confirmed the hypercellular and disrupted architecture in the marrow and spleen and concomitant extramedullary hematopoiesis. ETV6::RUNX1 overexpression managed to increase endogenous *Igf2bp1* levels in the bone marrow thus providing proof of our *in-vitro* finding.

Overexpression of ETV6::RUNX1 has been shown to increase HSCs with increased quiescence. Mature B-cell output has been known to be decreased with a developmental block at the pro-B cell stage [10, 65]. In our combination experiments, Hardy fraction analysis showed an expansion of the small and large pre-B cell fractions indicating a developmental arrest. It is interesting to speculate a co-operative role for ETV6::RUNX1 and IGF2BP1 where the ETV6::RUNX1 promotes the progenitor expansion and prevents B-cell maturation which are then driven towards further expansion and leukemogenesis by the pro-oncogenic pathway genes stabilized by IGF2BP1.

Although the mice did not develop overt leukemia by 16 weeks, the observed features appear to mimic a pre-leukemic phenotype. Our findings appear to agree with previous studies which show that secondary hits are necessary for leukemogenesis even after ETV6::RUNX1 overexpression in mouse HSCs [11]. Due to its weak oncogenic nature, only a fraction of children with the ETV6::RUNX1 translocation go on to develop B-ALL. The positive feedback shown between ETV6::RUNX1 and IGF2BP1 may develop over time, leading to an incrementally altered transcriptome and the eventual emergence of a dominant transformed clone during leukemogenesis. By enforcing IGF2BP1 expression, we attempted to accelerate this process; and our data bears out this additive/synergistic relationship.

## Conclusion

**Fig. 9:**
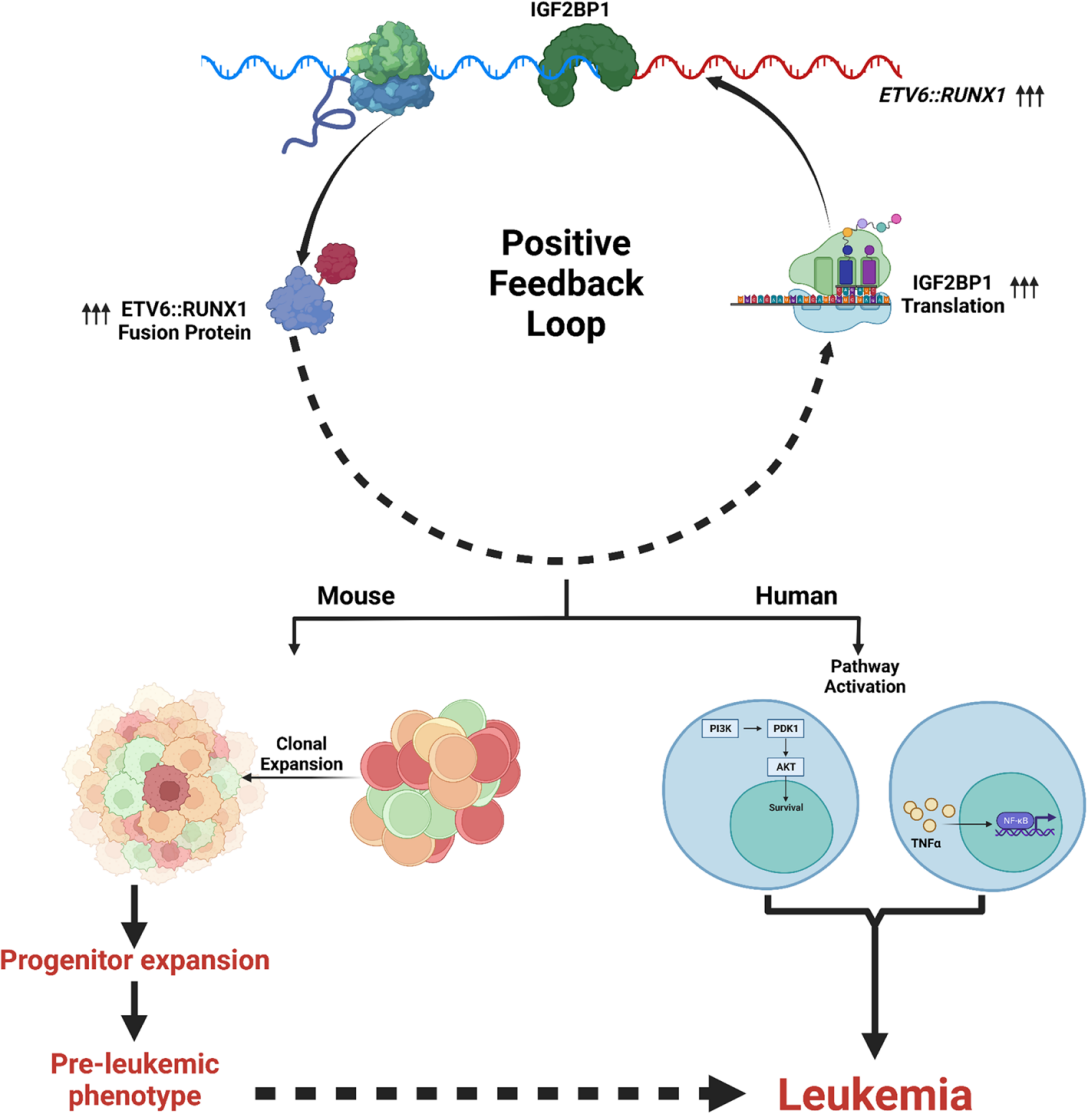
Graphical Abstract: Schematic depicting the synergism between IGF2BP1 and ETV6::RUNX1 to Drive Leukemogenesis in B-ALL. IGF2BP1 is overexpressed in ETV6::RUNX1 positive B-ALL cells and it regulates the expression of multiple pro-oncogenic signaling pathways, especially the TNFα/NFκB and PI3K-Akt pathways, by binding and stabilizing its target transcripts. IGF2BP1 also binds and stabilizes the ETV6::RUNX1 fusion transcript, forming a positive feedback loop. Co-expression of ETV6::RUNX1 and IGF2BP1 in murine bone marrow led to clonal expansion of immature progenitors and bone marrow hypercellularity. All these suggest that IGF2BP1 synergizes with ETV6::RUNX1 to drive leukemogenesis in this subtype of B-ALL

Our work uncovers a posttranscriptional, pro-oncogenic program driven by IGF2BP1 in *ETV6::RUNX1* positive B-ALL. At the functional level, we have characterized and validated the differential activity of the non-canonical NFκB and PI3K-Akt pathways. These pathways are also reinforced by the feedback between ETV6::RUNX1 and IGF2BP1 (Fig. 9). With many of these pathways being druggable and a small molecule inhibitor for IGF2BP1 available, our work lays the foundation for novel combinatorial therapeutic approaches in *ETV6::RUNX1* positive leukemias.

### Supplementary Information

**Additional file 1: Figure S1.** Relative Expression of ETV6-RUNX1 in multiple ETV6:: RUNX1 positive and negative cell lines. POLR2A and HGPRT were used as internal controls. **Figure S2.** SETEN analysis of RIP-Seq targets of IGF2BP1 using the A) MALA Cards B) GO Biological Processes and C) GO Molecular Function databases D) UCSC Genome browser snapshots of CDK6, MDM2, IL6ST, NFAT5 showing the enrichment of these targets in the IGF2BP1 precipitated fraction. **Figure S3.** GSEA (Hallmark pathways) of differentially expressed genes between ETV6::RUNX1 positive (*n*=14) and TCF3::PBX1 positive (*n*=15) subtypes of B-ALL patient samples showing activated and suppressed pathways with enrichment scores. **Figure S4.** GSEA (Hallmark pathways) of differentially expressed genes between ETV6::RUNX1 positive (*n*=14) and KMT2A translocated (*n*=15) subtypes of B-ALL patient samples showing activated and suppressed pathways with enrichment scores. **Figure S5.** A comparison of selected genes’ expression in the TNFα via NFκB and PI3K-AKT pathways from the a) IGF2BP1 knockout dataset (log2Fc; <1 refers to downregulation) b and c) analysis of public microarray based dataset of ETV6::RUNX1 vs TCF3::PBX1 patient samples and ETV6::RUNX1 vs KMT2A rearranged patient samples (log2Fc) d) RIP enrichment based on fold change of IGF2BP1 immunoprecipitated RNA to input RNA levels and e) public dataset of differentially expressed genes between ETV6::RUNX1 positive B-ALL patients (*n*=4) and CD19 positive B-cells (*n*=2) (log2Fc). The TNFα via NFκB pathway targets are downregulated after IGF2BP1 KO, enriched in the RIP data and the ETV6::RUNX1 positive tumors showing that these are direct targets bound and stabilized by IGF2BP1. The PI3K pathway targets are bound by IGF2BP1 and enriched in the tumors but not downregulated after IGF2BP1 KO implying a multifactorial regulation of their mRNA stability. **Figure S6.** A) Validation of RIP targets of IGF2BP1 using the public TARGET dataset (E6R1+ *n*=20, Other translocations *n*=27, Hyperdiploid *n*=55, No Known Sentinel Translocations *n*=95) B) Correlation between IGF2BP1 and its target genes in the patient samples with high IGF2BP1 expression (*n*=29). **Figure S7.** A)-B) Correlation between IGF2BP1 and its target genes in the TARGET cohort (*n*=29) C) Expression of BCL2 and MYB after IGF2BP1 KO and their RIP enrichment from our datasets. **Figure S8.** Cell viability in (A-D) ETV6-RUNX1 translocation positive cell lines along with E) RL (Non-Hodgkin lymphoma) and F) Jurkat, a T-ALL cell line after functional inhibition of IGF2BP1 using BTYNB treatment. Cell viability measured by MTS assay (AC) and trypan blue exclusion assay (D-F). **Figure S9.** Expression of IGF2BP1 and ETV6::RUNX1 in ETV6-RUNX1 translocation positive cell lines as determined by qRT-PCR after BTYNB treatment (5 µM). **Figure S10.** Expression of IGF2BP1 targets of NFKB and PI3K-AKT signalling pathway after IGF2BP1 inhibition by BTYNB (5/10µM) treatment compared to DMSO-treated cells. (AT1, AT2, UOCB6 – ETV6-RUNX1 translocation positive cell lines and RS4;11, NALM6, THP1 – ETV6-RUNX1 translocation negative cell lines). POLR2A and HGPRT were used as internal controls. **Figure S11.** Western Blots showing reduced protein expression of IGF2BP1 targets of NFkB (IKKα and NFκβ) and PI3K-AKT (AKT, MDM2, GSK3β and PDK1) signalling pathway in ETV6-RUNX1 positive cell lines after IGF2BP1 inhibition by BTYNB. Densitometric values are provided above each blot normalized to the DMSO control. GAPDH was used as internal control. (Note: For NFkB, densitometry was performed using p50, the functional sub-unit.). **Figure S12.** Figure S11: Western Blots showing no change in protein expression of IGF2BP1 targets of NFkB (IKKα and NFκβ) and PI3K-AKT (AKT, MDM2, GSK3β and PDK1) signalling pathway in ETV6-RUNX1 positive cell lines after IGF2BP1 inhibition by BTYNB. Densitometric values are provided above each blot normalized to the DMSO control. GAPDH was used as internal control. (Note: For NFkB, densitometry was performed using p50, the functional sub-unit; RS4;11 expressed extremely low levels of p-GSK3β (Ser9) to detect and analyze). **Figure S13.** A) Schematic of cloning the IGF2BP1 CDS in a pHAGE6 based lentiviral vector and cloning the ETV6::RUNX1 fusion junction in a dual luciferase pMirGlo vector B) Schematic showing regions targeted by ETV6::RUNX1 targeted guide RNAs which were then cloned in pLKO5-tRFP vector C) Schematic of ETV6::RUNX1 fusion transcript overexpressing retroviral vector. **Figure S14.** A) Schematic of the bone marrow transplant experiment B) Vector schematic of the ETV6::RUNX1 fusion transcript and IGF2BP1 overexpressing murine retroviral vectors C) Western blot showing overexpression of IGF2BP1 in 293T cells after MICHIGF2BP1 transfection; GAPDH was used as a loading control D) qRT-PCR to quantify overexpression of ETV6::RUNX1 in 293T cells after MIG-ETV6::RUNX1 transfection. **Figure S15.** A) Time course graph showing increasing clonal expansion in the peripheral blood of IGF2BP1+ETV6::RUNX1 combination group with time (t-test; *p* * < 0.05, ** < 0.01, *** < 0.005, **** < 0.0001) B) Representative FACS plots indicating clonal expansion. **Figure S16.** A-H) Quantification of various hematological parameters from the peripheral blood of mice at 16 weeks (t-test; *p* * < 0.05, ** < 0.01, *** < 0.005, **** <0.0001). **Figure S17.** Quantification of immature B-cell populations (Hardy Fractions) in the bone marrow (absolute counts and percentages) (t-test; *p* * < 0.05, ** < 0.01, *** < 0.005, **** <0.0001). **Figure S18.** Quantification of absolute counts of myeloid lineage committed progenitors (CMP, GMP and MEP) and their respective percentages in the bone marrow (t-test; *p* *< 0.05, ** < 0.01, *** < 0.005, **** <0.0001). **Figure S19.** Subset analysis of various progenitor populations in different fractions of the bone marrow: GFP+mCherry+ (double positive) and GFP-mCherry- (double negative) fractions; This revealed a cell intrinsic and extrinsic role in the IGF2BP1+ETV6::RUNX1 combination group favoring progenitor expansion (t-test; *p* *<0.05, ** < 0.01, *** < 0.005, **** < 0.0001). **Figure S20.** A) Spleen weights of mice belonging to different groups at week 16 B) Quantification of double positive ratio in the splenocytes and C) Lin- population by the ratio of GFP+ mCherry+ double positive cells D) Quantification of HSCs in the spleen (t-test; *p* *< 0.05, ** < 0.01, *** < 0.005, **** < 0.0001) E) Histological analysis of spleens of mice belonging to different groups; Controls show a prominent germinal center and marginal zone with clear white and red pulp distinction; Red pulp expansion, smaller germinal centers and loss of architecture is seen in IGF2BP1+ETV6::RUNX1 combination (200X). **Supplementary Table 1.** List of primers used in this study. **Supplementary Table 2.** List of guide RNAs used in this study. **Supplementary Table 3.** Surface markers for hematopoietic progenitors. **Supplementary Table 4.** Hardy fractions Surface markers. **Supplementary Table 5.** List of antibodies used in flowcytometry.

**Additional file 2. **

## Data Availability

The analyzed RNA-Seq and RIP-Seq data can be found in a data supplement available with the online version of this article. Raw reads are available in the SRA database with BioProject ID PRJNA837729. We have utilized some public ALL transcriptomic datasets to corroborate our data from the cBioportal (https://www.cbioportal.org/). The results published here are in whole or part based upon data generated by the Therapeutically Applicable Research to Generate Effective Treatments (https://ocg.cancer.gov/programs/target) initiative, phs000463 and phs000464. The data used for this analysis are available at ‘https://portal.gdc.cancer.gov/projects’

## Abbreviations

IGF2BP1: Insulin like Growth Factor 2 Binding Protein 1
ALL: Acute Lymphoblastic Leukemia
RIP-Seq: RNA Immunoprecipitation sequencing
LSC: Leukemic Stem Cell
OS: Overall survival
EFS: Event free survival
sgRNA: single guide RNA
GSEA: Gene Set Enrichment Analysis
TARGET: Therapeutically Applicable Research to Generate Effective Treatments
MSCV: Murine Stem Cell Virus
MICH: MSCV-IRES-mCherry
MIG: MSCV-IRES-GFP
HSC: Hematopoietic Stem Cells
LMPP: Lymphomyeloid Primed Progenitors
CLP: Common Lymphoid Progenitors
LSK: Lin- Sca1+ cKit+
CMP: Common Myeloid Progenitors
GMP: Granulocyte-Monocyte Progenitor
MEP: Megakaryocyte-Erythroid Progenitor
ICiCLe: Indian Childhood Collaborative Leukemia Group
