# Supplementary material for: RNA binding protein IGF2BP1 synergizes with ETV6-RUNX1 to drive oncogenic signaling in B-cell Acute Lymphoblastic Leukemia
Source: J Exp Clin Cancer Res. 2023 Sep 5;42:231. doi: 10.1186/s13046-023-02810-1 (PMC10478443; doi:10.1186/s13046-023-02810-1)
Supplement: Supplementary file 1 — Additional file 1: Figure S1. Relative Expression of ETV6-RUNX1 in multiple ETV6:: RUNX1 positive and negative cell lines. POLR2A and HGPRT were used as internal controls. Figure S2. SETEN analysis of RIP-Seq targets of IGF2BP1 using the A) MALA Cards B) GO Biological Processes and C) GO Molecular Function databases D) UCSC Genome browser snapshots of CDK6, MDM2, IL6ST, NFAT5 showing the enrichment of these targets in the IGF2BP1 precipitated fraction. Figure S3. GSEA (Hallmark pathways) of differentially expressed genes between ETV6::RUNX1 positive (n=14) and TCF3::PBX1 positive (n=15) subtypes of B-ALL patient samples showing activated and suppressed pathways with enrichment scores. Figure S4. GSEA (Hallmark pathways) of differentially expressed genes between ETV6::RUNX1 positive (n=14) and KMT2A translocated (n=15) subtypes of B-ALL patient samples showing activated and suppressed pathways with enrichment scores. Figure S5. A comparison of selected genes’ expression in the TNFα via NFκB and PI3K-AKT pathways from the a) IGF2BP1 knockout dataset (log2Fc; <1 refers to downregulation) b and c) analysis of public microarray based dataset of ETV6::RUNX1 vs TCF3::PBX1 patient samples and ETV6::RUNX1 vs KMT2A rearranged patient samples (log2Fc) d) RIP enrichment based on fold change of IGF2BP1 immunoprecipitated RNA to input RNA levels and e) public dataset of differentially expressed genes between ETV6::RUNX1 positive B-ALL patients (n=4) and CD19 positive B-cells (n=2) (log2Fc). The TNFα via NFκB pathway targets are downregulated after IGF2BP1 KO, enriched in the RIP data and the ETV6::RUNX1 positive tumors showing that these are direct targets bound and stabilized by IGF2BP1. The PI3K pathway targets are bound by IGF2BP1 and enriched in the tumors but not downregulated after IGF2BP1 KO implying a multifactorial regulation of their mRNA stability. Figure S6. A) Validation of RIP targets of IGF2BP1 using the public TARGET dataset (E6R1+ n=20, Other translocations n=27, [file 13046_2023_2810_MOESM1_ESM.pdf]

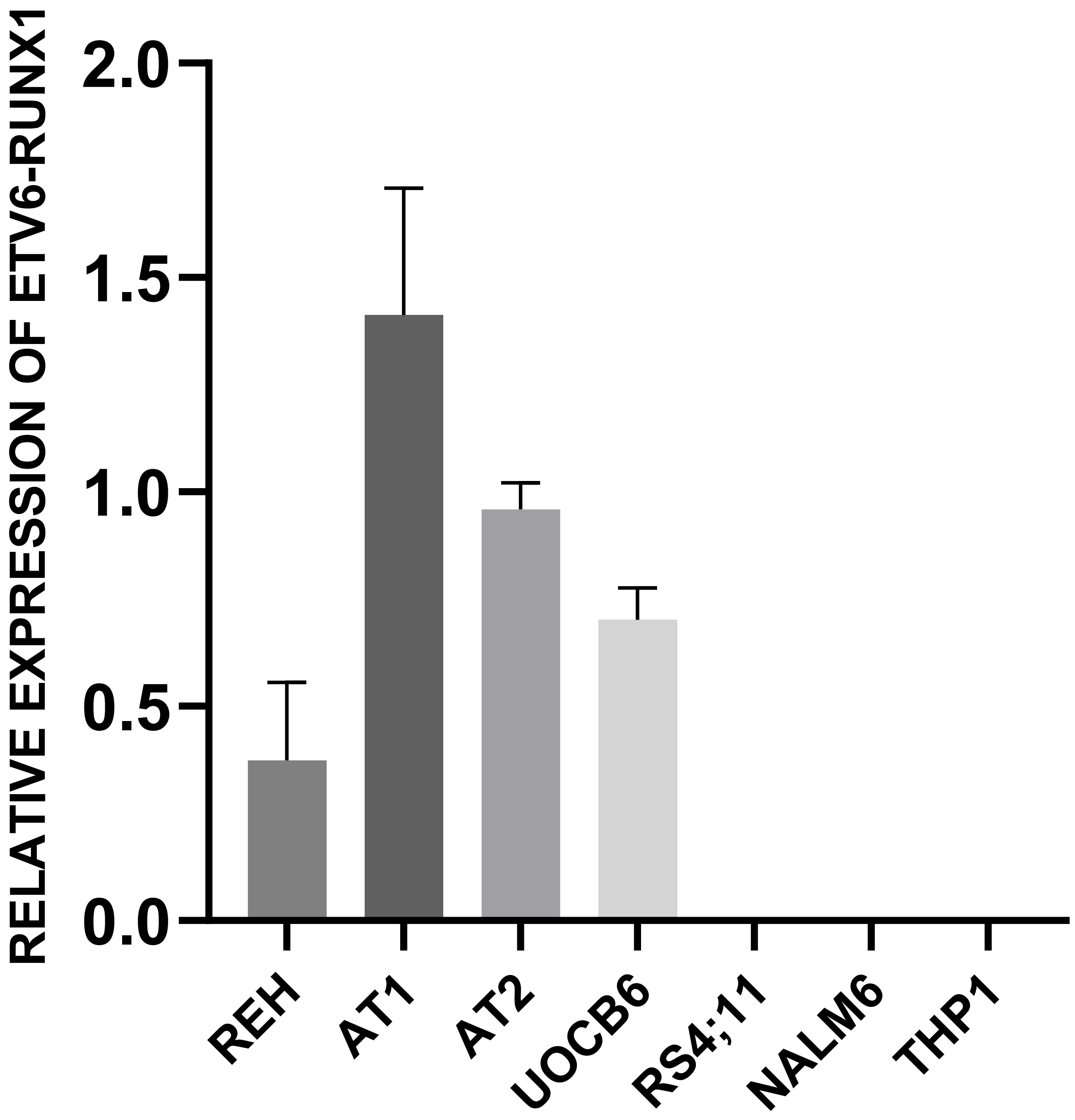

Figure S1: Relative Expression of *ETV6::RUNX1* in multiple *ETV6::RUNX1* positive and negative cell lines.
POLR2A and *HGPRT* : Housekeeping genes.

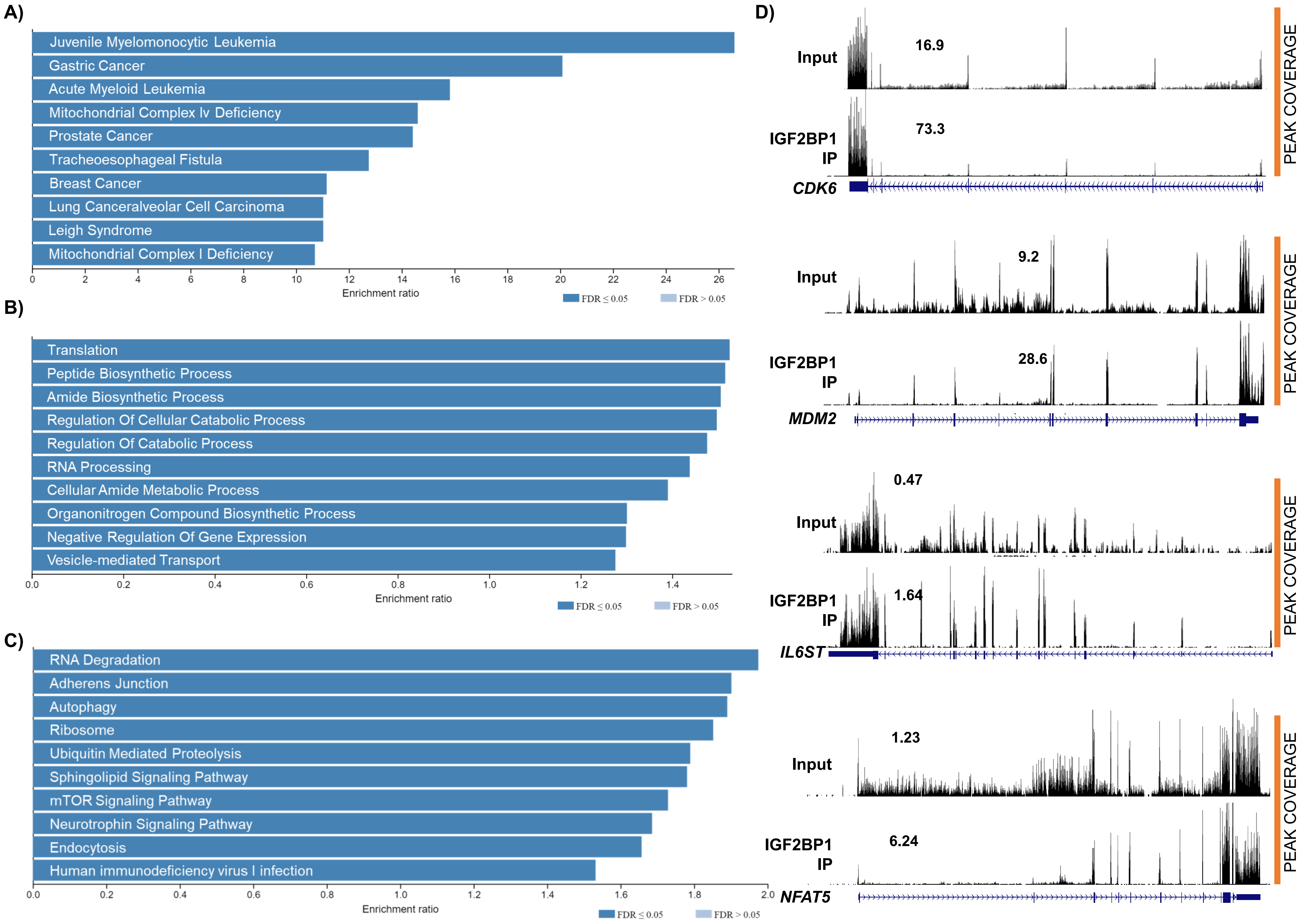

Figure S2: SETEN analysis of RIP-Seq targets of IGF2BP1 using the A) MALA Cards B) GO Biological Processes and C) GO Molecular Function databases D) UCSC Genome browser snapshots of *CDK6, MDM2, IL6ST, NFAT5* showing the enrichment of these targets in the IGF2BP1 precipitated fraction

ETV6::RUNX1 VS TCF3::PBX1

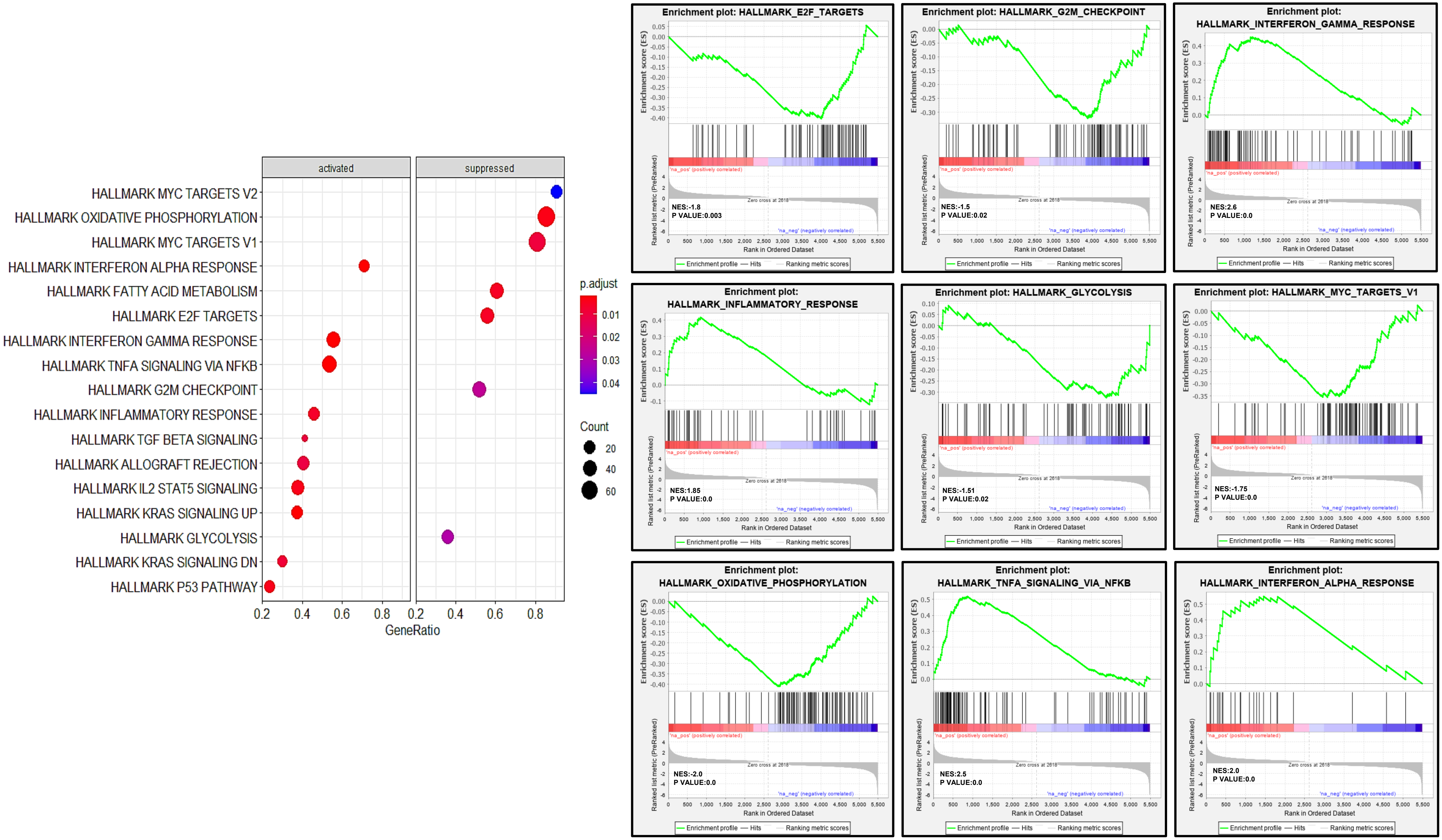

Figure S3: GSEA (Hallmark pathways) of differentially expressed genes between *ETV6::RUNX1* positive (n=14) and *TCF3::PBX1* positive (n=15) subtypes of B-ALL patient samples showing activated and suppressed pathways with enrichment scores

ETV6::RUNX1 VS KMT2A

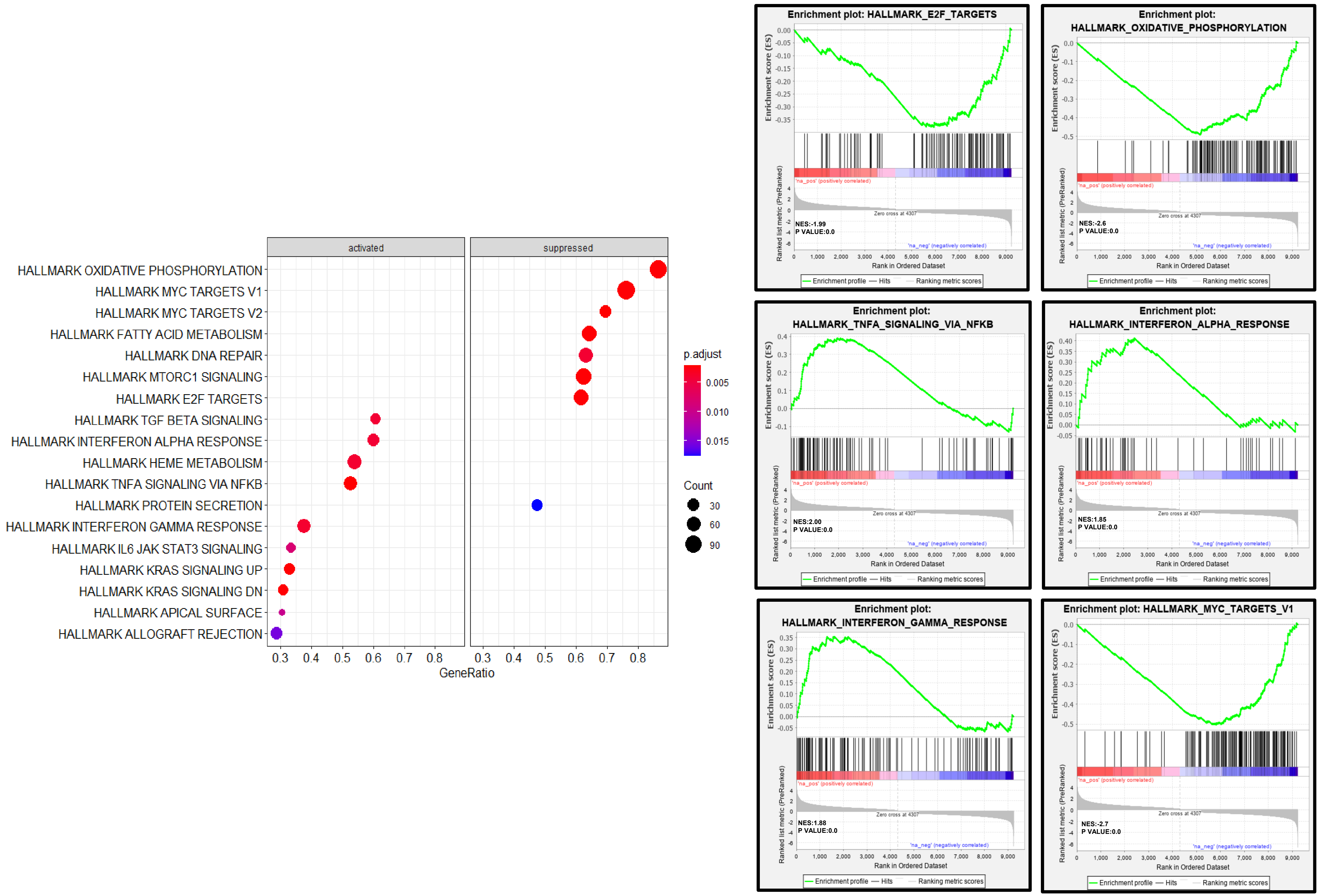

Figure S4: GSEA (Hallmark pathways) of differentially expressed genes between *ETV6::RUNX1* positive (n=14) and *KMT2A* translocated (n=15) subtypes of B-ALL patient samples showing activated and suppressed pathways with enrichment scores

TNFα VIA NFKB	IGF2BP1 KO	E6R1 VS TCF3::PBX1	E6R1 VS KMT2A	RIP Enrichment	E6R1 VS CD19+
**NFKB2**	0.65	1.34	1.55	+++	-2.74
**GADD45A**	0.52	2.60	3.08	++	7.50
**IL6ST**	0.68	1.83	1.85	++	5.29
**NFAT5**	0.85	1.94	NS	+	1.75

PI3K-Akt signaling pathway	IGF2BP1 KO	E6R1 VS TCF3::PBX1	E6R1 VS KMT2A	RIP Enrichment	E6R1 VS CD19+
**NGFR**	0.72	3.43	3.27	++	**30.60**
**FGFR1**	1.23	1.63	6.33	++	**25.22**
**MDM2**	1.34	2.42	2.68	++	**10.47**
**CDK6**	1.71	2.02	0.35	++++	**6.64**
**CCND1**	1.00	1.47	1.48	++	**2.70**

Figure S5: A comparison of selected genes' expression in the TNFα via NFκB and PI3K-AKT pathways from the a) *IGF2BP1* knockout dataset (log2Fc; <1 refers to downregulation) b and c) analysis of public microarray based dataset of *ETV6::RUNX1* vs *TCF3::PBX1* patient samples and *ETV6::RUNX1* vs *KMT2A* rearranged patient samples (log2Fc) d) RIP enrichment based on fold change of IGF2BP1 immunoprecipitated RNA to input RNA levels and e) public dataset of differentially expressed genes between *ETV6::RUNX1* positive B-ALL patients (n=4) and CD19 positive B-cells (n=2) (log2Fc). The TNFα via NFκB pathway targets are downregulated after IGF2BP1 KO, enriched in the RIP data and the *ETV6::RUNX1* positive tumors showing that these are direct targets bound and stabilized by IGF2BP1. The PI3K pathway targets are bound by IGF2BP1 and enriched in the tumors but not downregulated after IGF2BP1 KO implying a multifactorial regulation of their mRNA stability.

A) NFκB SIGNALLING PATHWAY

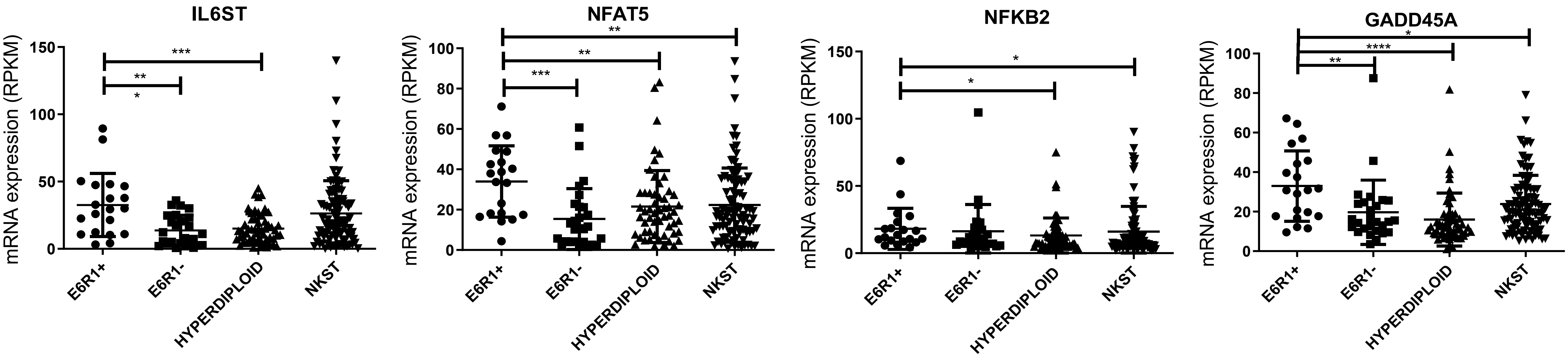

B) PI3K-AKT SIGNALLING PATHWAY

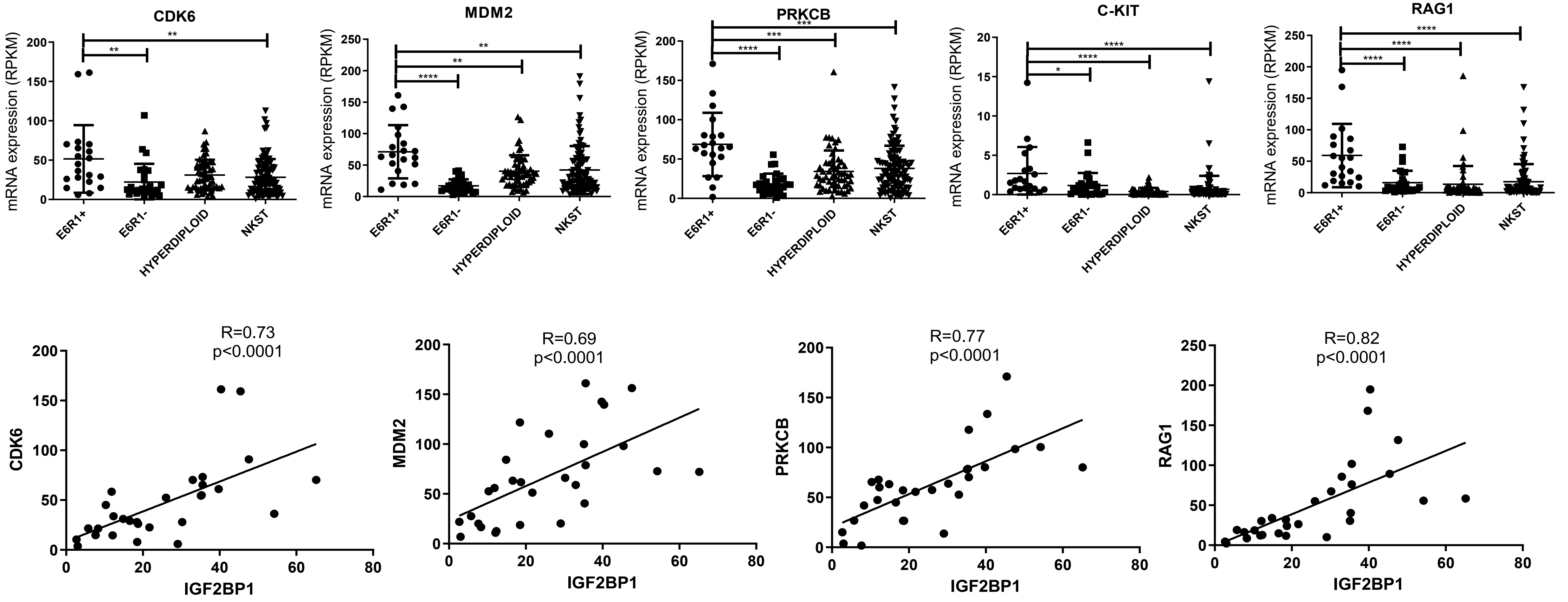

Figure S6: A) Validation of RIP targets of IGF2BP1 using the public TARGET dataset (E6R1+ n=20, Other translocations n=27, Hyperdiploid n=55, No Known Sentinel Translocations n=95) B) Correlation between IGF2BP1 and its target genes in the patient samples with high IGF2BP1 expression (n=29)

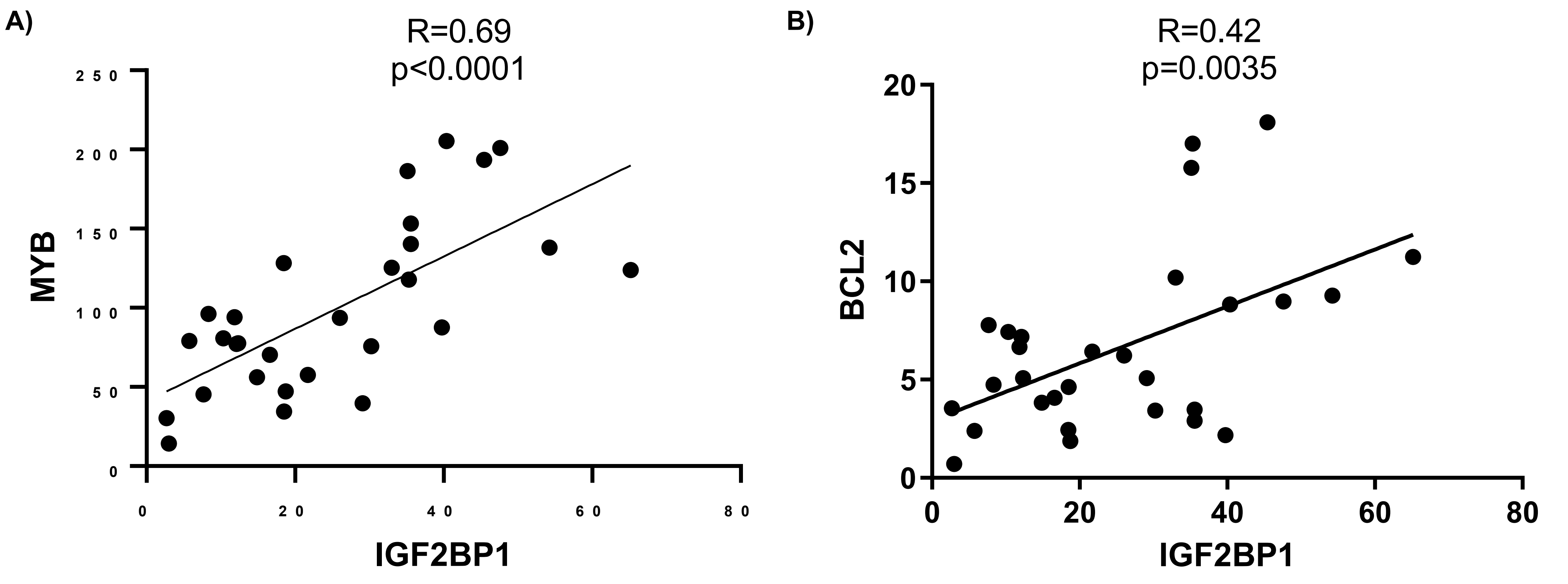

C)

PROPERTIES	GENE NAME	Log FC	p-value	FDR	RIP ENRICHMENT
**GLUCOCORTICOID RESISTANCE (Zhang et al)**	BCL2	-0.57499	4.01E-27	1.14E-24	YES
**STEMNESS (Elcheva et al)**	MYB	-0.34044	4.73E-12	2.73E-10	YES

Figure S7 A)-B) Correlation between IGF2BP1 and its target genes in the TARGET cohort (n=29) C) Expression of BCL2 and MYB after IGF2BP1 KO and their RIP enrichment from our datasets

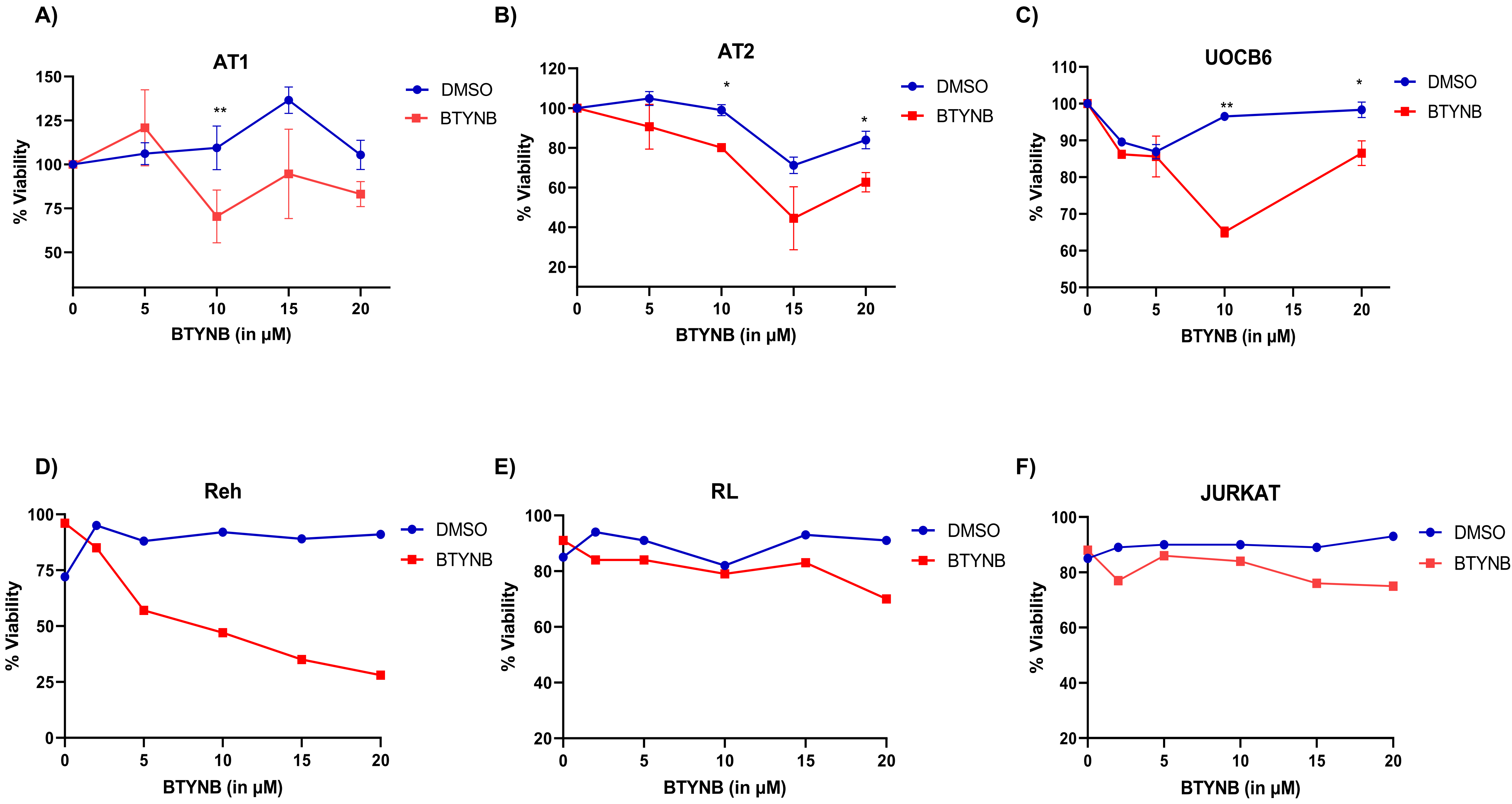

Figure S8: Cell viability in (A-D) *ETV6::RUNX1* translocation positive cell lines along with E) RL (Non-Hodgkin lymphoma) and F) Jurkat, a T-ALL cell line after functional inhibition of IGF2BP1 using BTYNB treatment. Cell viability measured by MTS Assay (A-C) and trypan blue exclusion assay (D-F).

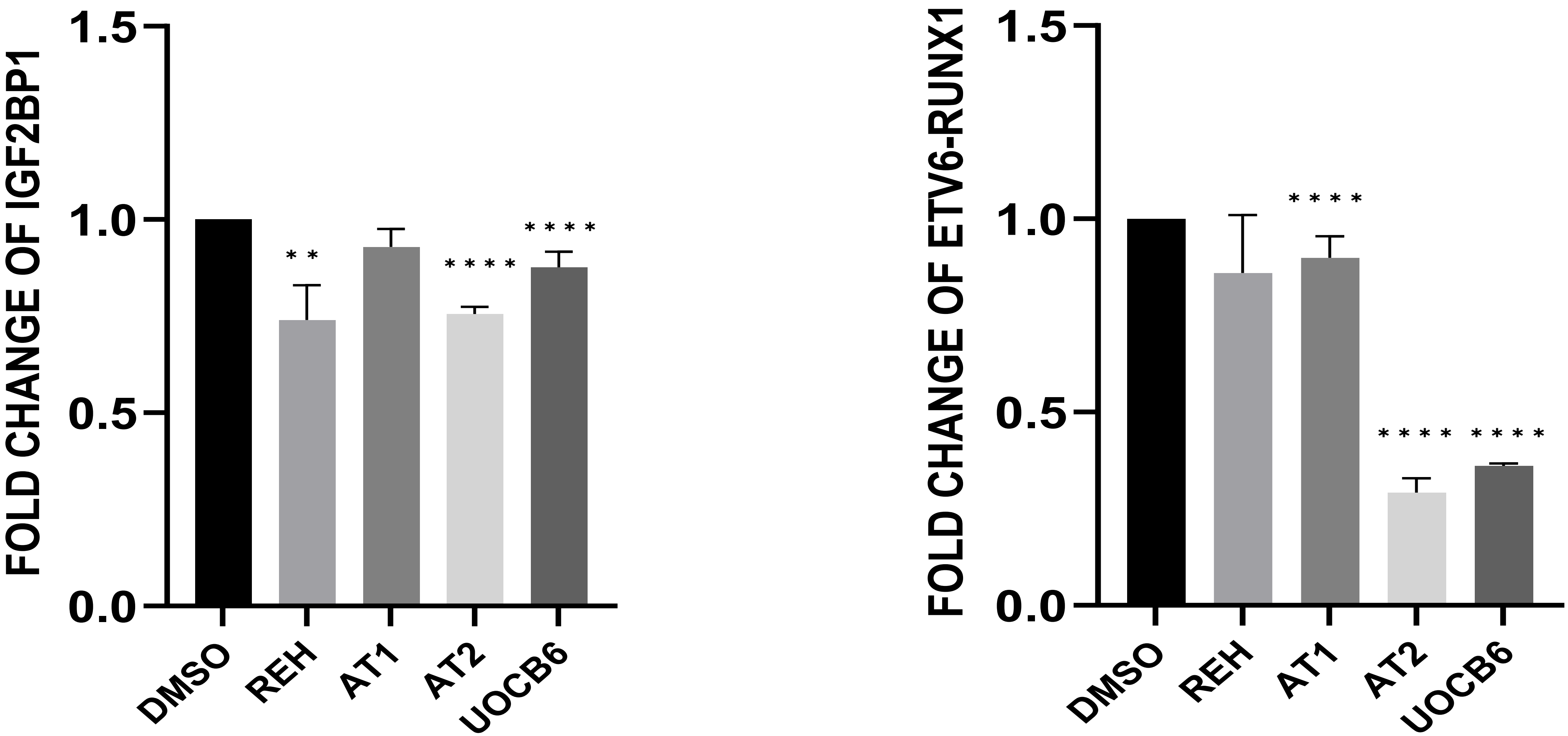

Figure S9: Expression of *IGF2BP1* and *ETV6::RUNX1* in *ETV6::RUNX1* translocation positive cell lines as determined by qRT-PCR after BTYNB treatment (5µM)

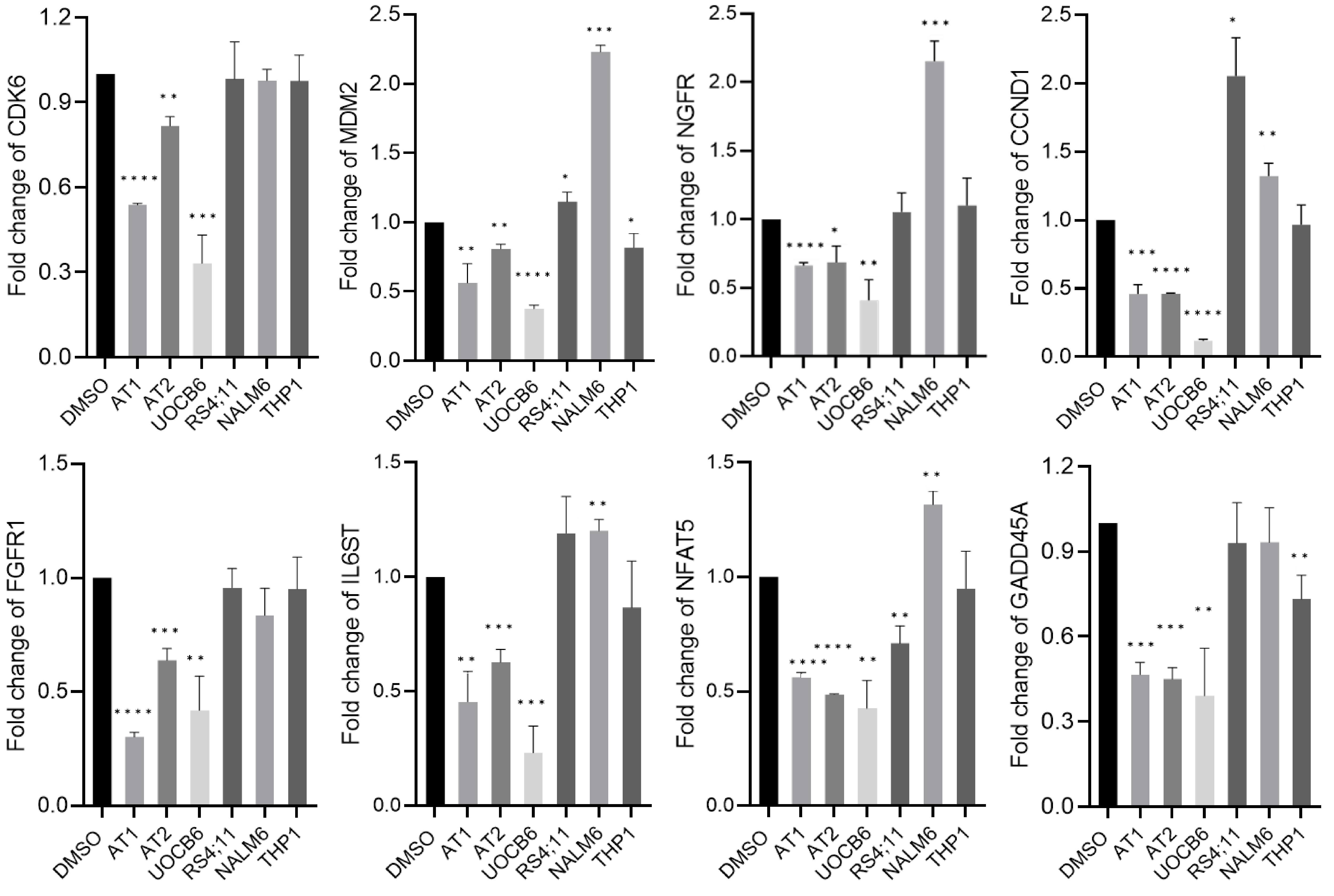

Figure S10: Expression of IGF2BP1 targets of NFKB and PI3K-AKT signalling pathway after *IGF2BP1* inhibition by BTYNB (5/10μM) treatment compared to DMSO-treated cells. (AT1, AT2, UOCB6 – *ETV6::RUNX1* translocation positive cell lines and RS4;11, NALM6, THP1 – *ETV6::RUNX1* translocation negative cell lines). *POLR2A* and *HGPRT*: Housekeeping genes. DMSO used as vehicle control.

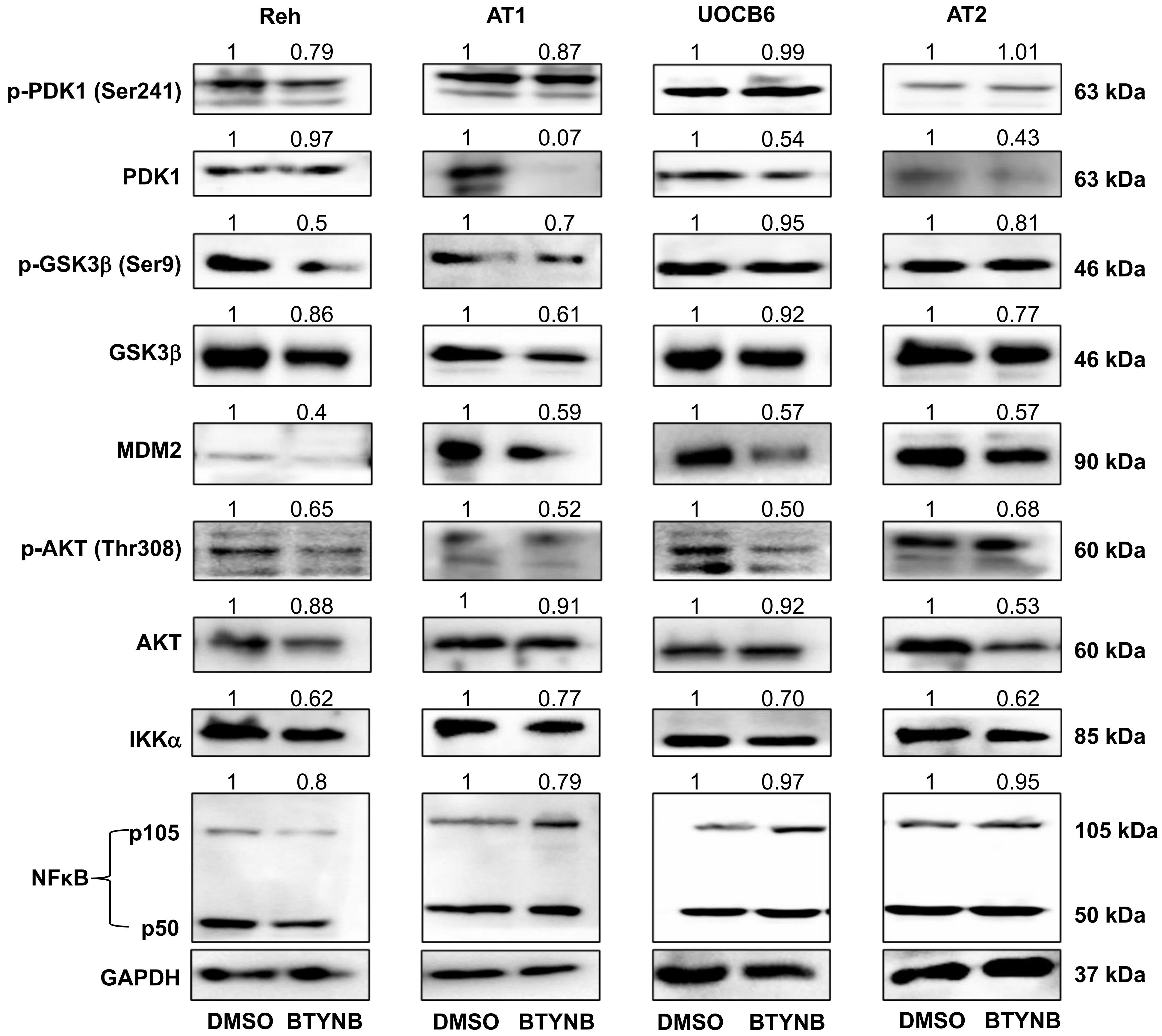

Figure S11: Western Blots showing reduced protein expressions of IGF2BP1 targets of NFkB (IKKα and NFκB) and PI3K-AKT (AKT, MDM2, GSK3β and PDK1) signalling pathway in *ETV6::RUNX1* positive cell lines after *IGF2BP1* inhibition by BTYNB treatment. Densitometric values are provided above each blot normalised to the DMSO control. GAPDH was used as internal control.(Note: For NFkB, densitometry was performed using p50, the functional sub-unit.).

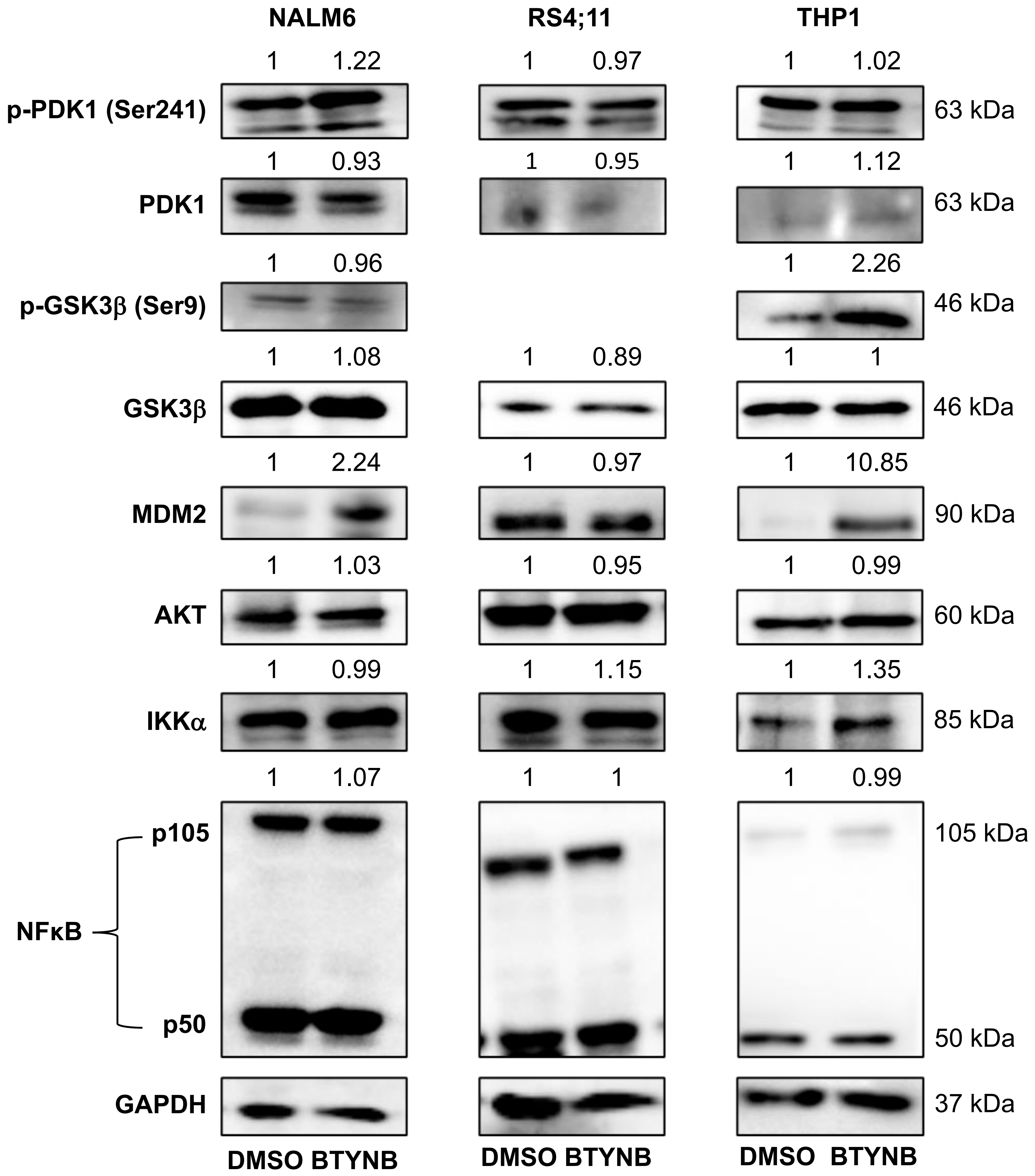

Figure S12: Western Blots showing no chage in protein expression of some of IGF2BP1 targets of NFkB (IKKα and NFκB) and PI3K-AKT (AKT, MDM2, GSK3β and PDK1) signalling pathway in *ETV6::RUNX1* translocation negative cell lines after *IGF2BP1* inhibition by BTYNB (5μM) treatment. Densitometric values are provided above each blot normalised to the DMSO control. (Note: For NFkB, densitometry was performed using p50, the functional sub-unit.; RS4;11 expressed extremely low levels of p-GSK3β (Ser-9) to detect and analyze) GAPDH: internal control.

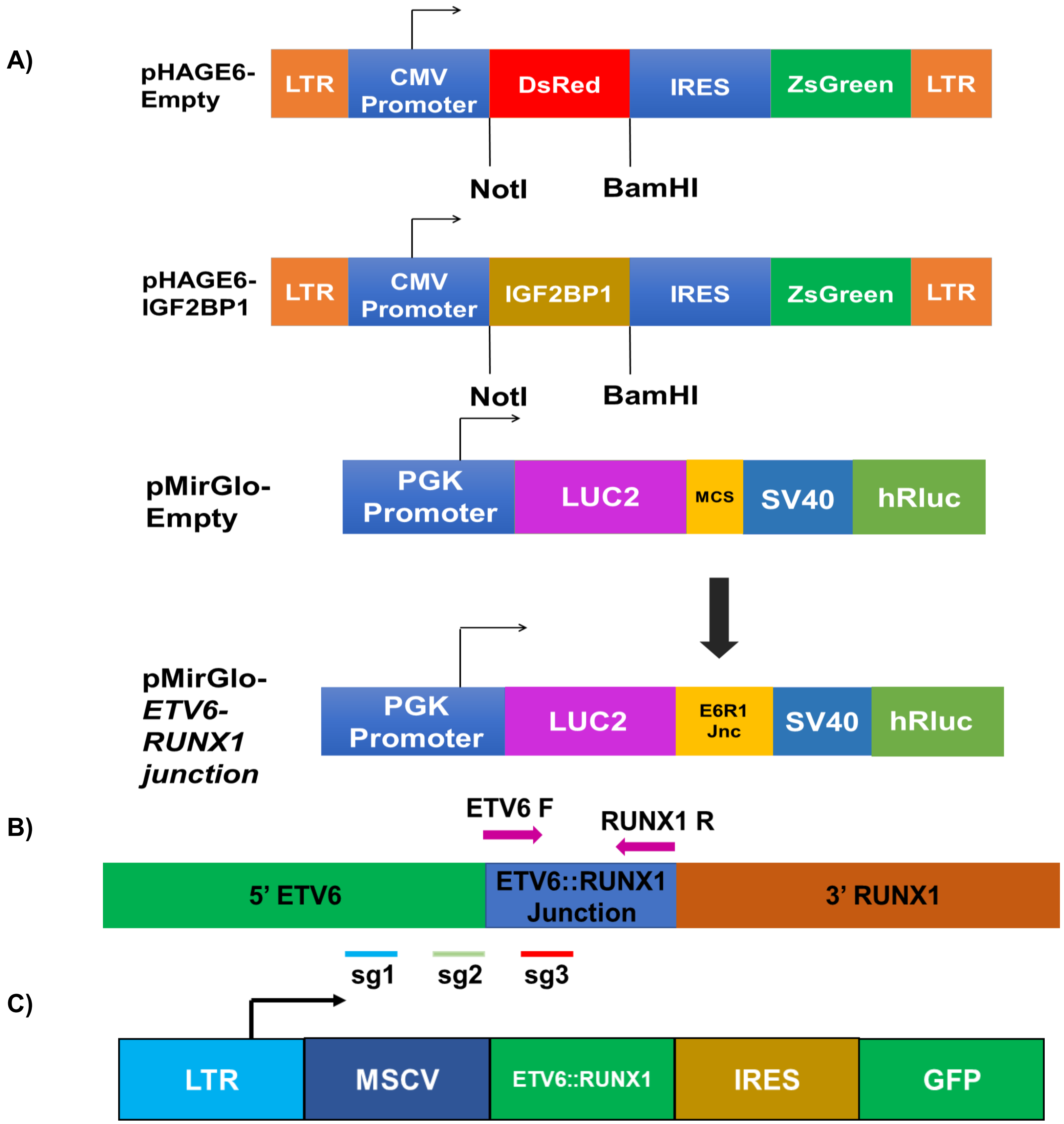

Figure S13: A) Schematic of cloning the *IGF2BP1* CDS in a pHAGE6 based lentiviral vector and cloning the *ETV6::RUNX1* fusion junction in a dual luciferase pMirGlo vector B) Schematic showing regions targeted by ETV6::RUNX1 targeted guide RNAs which were then cloned in pLKO5-tRFP vector C) Schematic of *ETV6::RUNX1* fusion transcript overexpressing retroviral vector

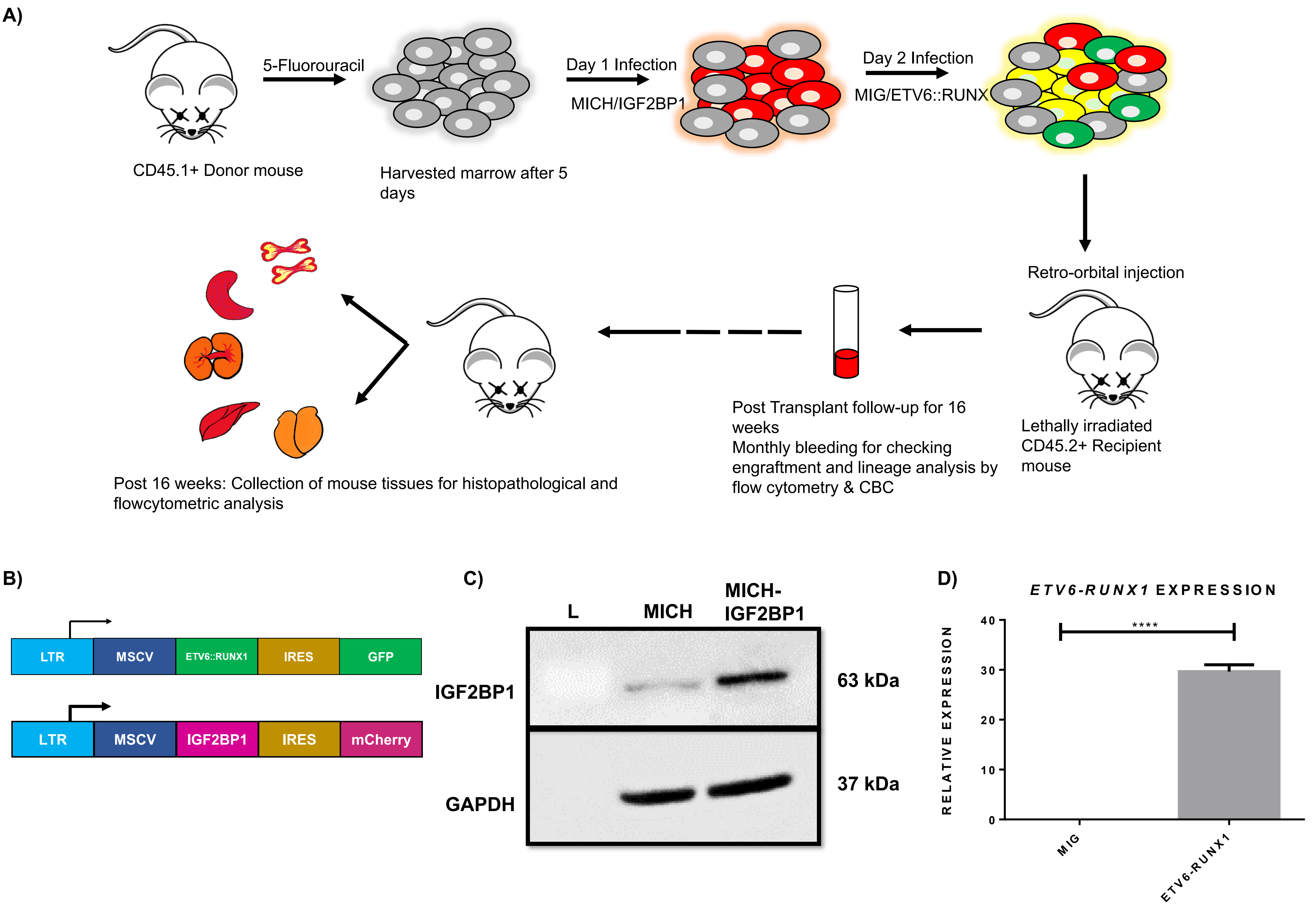

Figure S14: A) Schematic of the bone marrow transplant experiment B) Vector schematic of the *ETV6::RUNX1* fusion transcript and *IGF2BP1* overexpressing murine retroviral vectors C) Western blot showing overexpression of IGF2BP1 in 293T cells after MICH-IGF2BP1 transfection; GAPDH was used as a loading control D) qRT-PCR to quantify overexpression of *ETV6::RUNX1* in 293T cells after MIG-ETV6::RUNX1 transfection

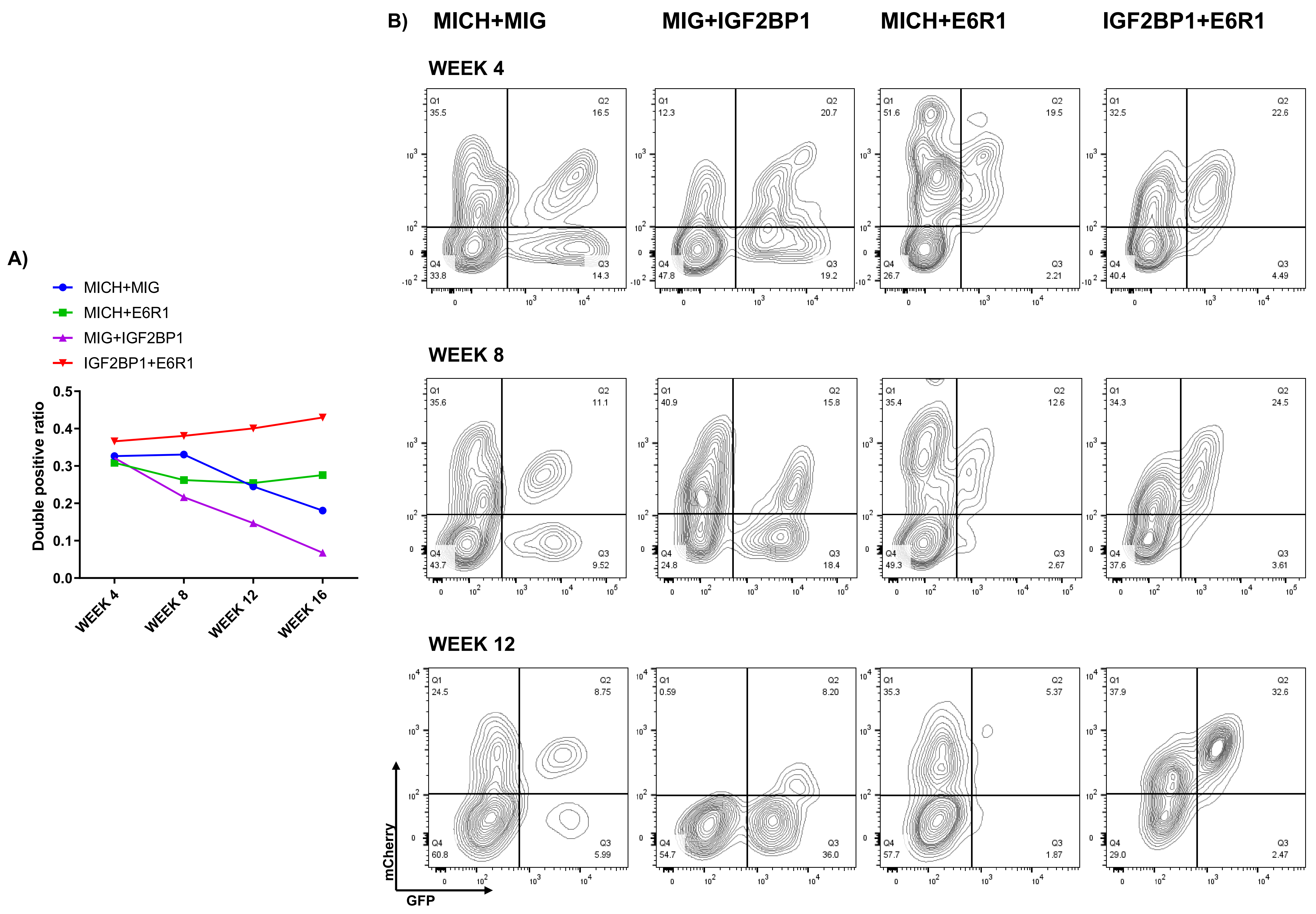

Figure S15: A) Time course graph showing increasing clonal expansion in the peripheral blood of IGF2BP1+ETV6::RUNX1 combination group with time (t-test; p * <0.05, ** <0.01, *** <0.005, **** <0.0001) B) Representative FACS plots indicating clonal expansion

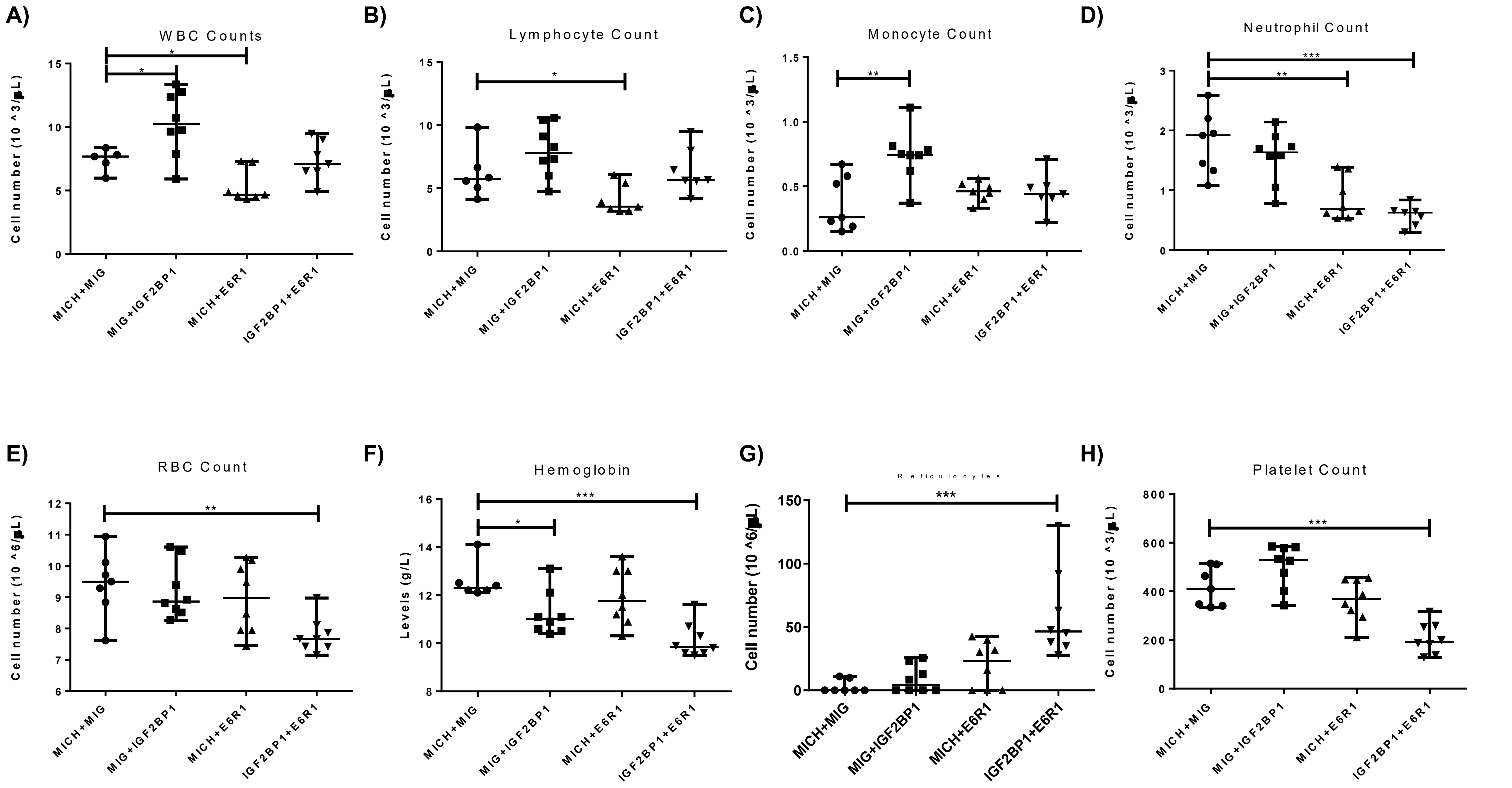

Figure S16: A-H) Quantification of various hematological parameters from the peripheral blood of mice at 16 weeks (t-test; p * <0.05, ** <0.01, *** <0.005, **** <0.0001).

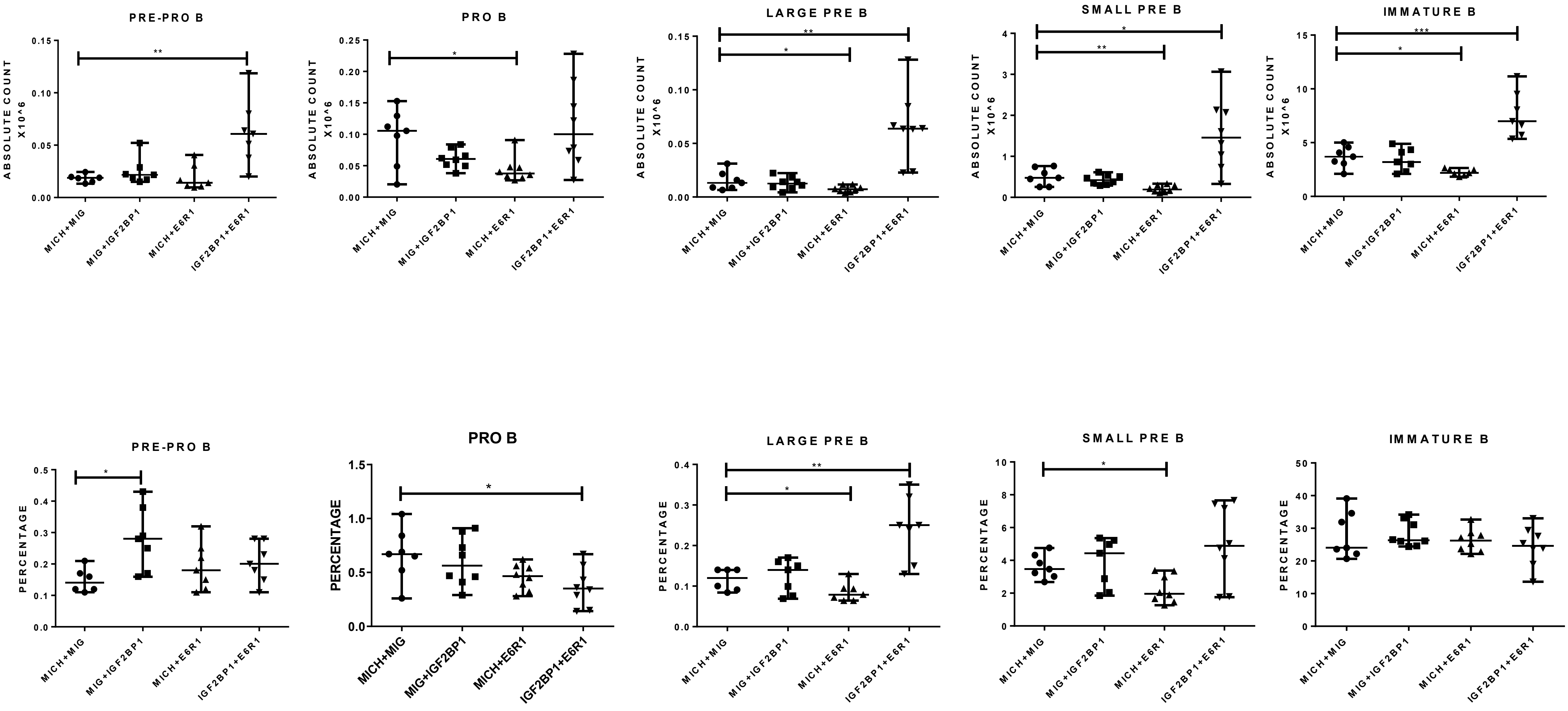

Figure S17: Quantification of immature B-cell populations (Hardy Fractions) in the bone marrow (absolute counts and percentages) (t-test; p * <0.05, ** <0.01, *** <0.005, **** <0.0001).

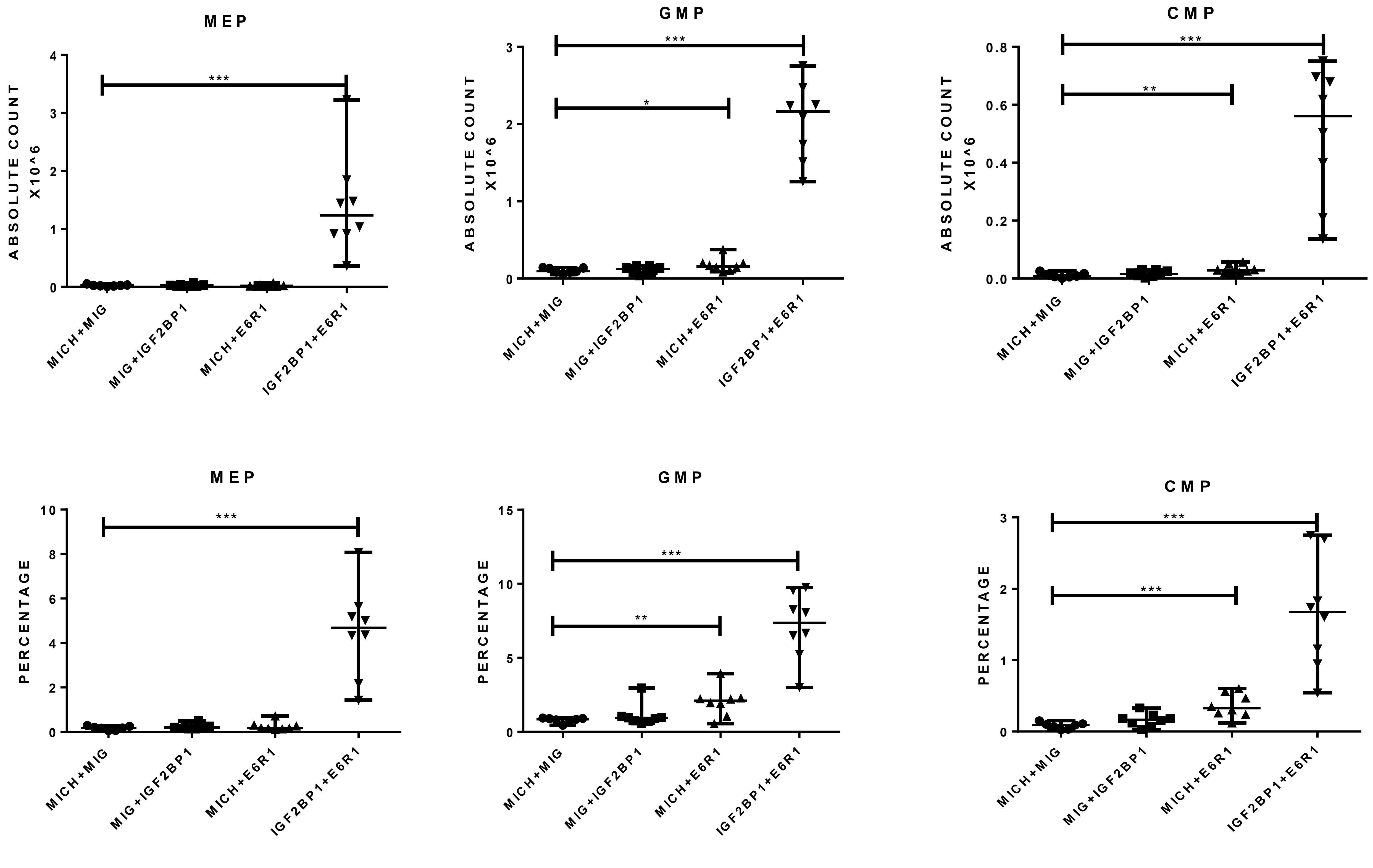

Figure S18: Quantification of absolute counts of myeloid lineage committed progenitors (CMP, GMP and MEP) and their respective percentages in the bone marrow (t-test; p *<0.05, ** <0.01, *** <0.005, **** <0.0001)

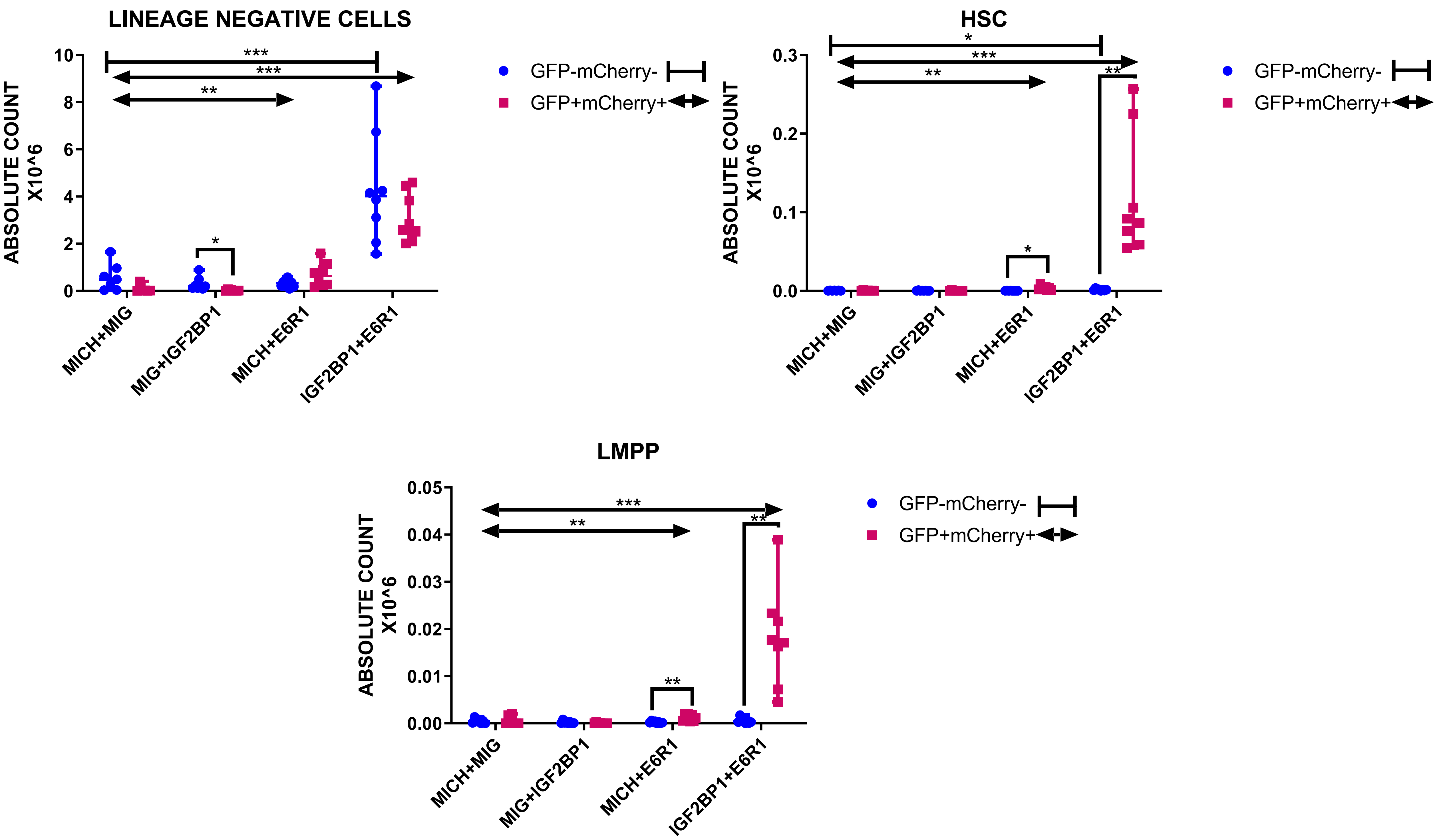

Figure S19: Subset analysis of various progenitor populations in different fractions of the bone marrow: GFP+mCherry+ (double positive) and GFP-mCherry- (double negative) fractions; This revealed a cell intrinsic and extrinsic role in the IGF2BP1+ETV6::RUNX1 combination group favoring progenitor expansion (t-test; p *<0.05, ** <0.01, *** <0.005, **** <0.0001)

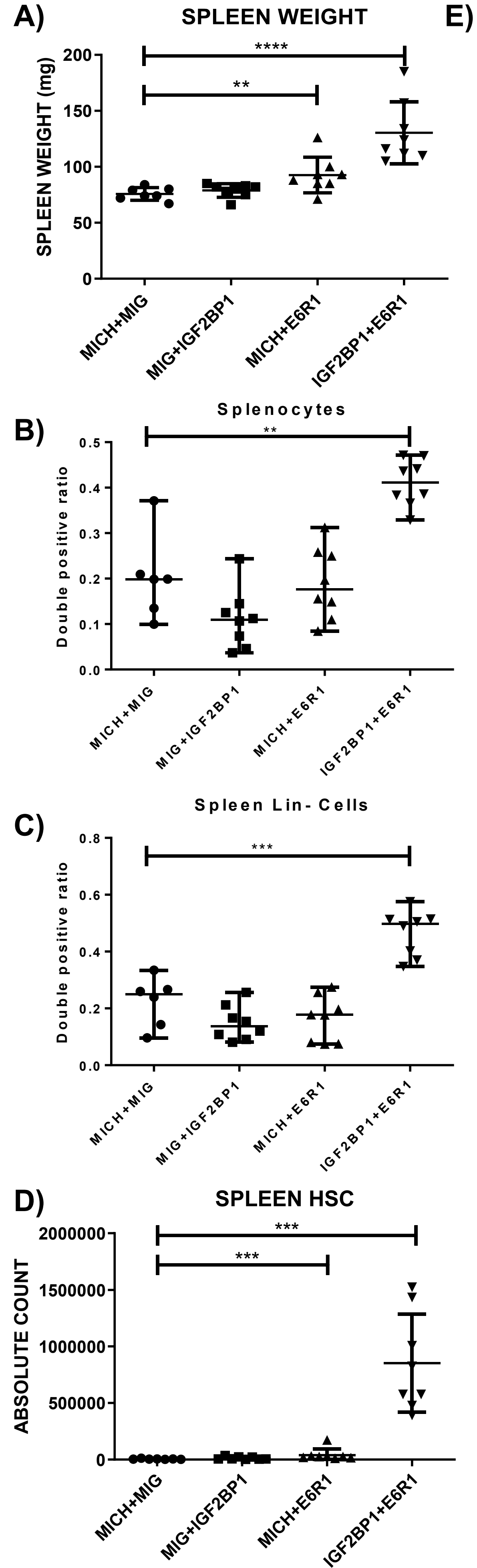

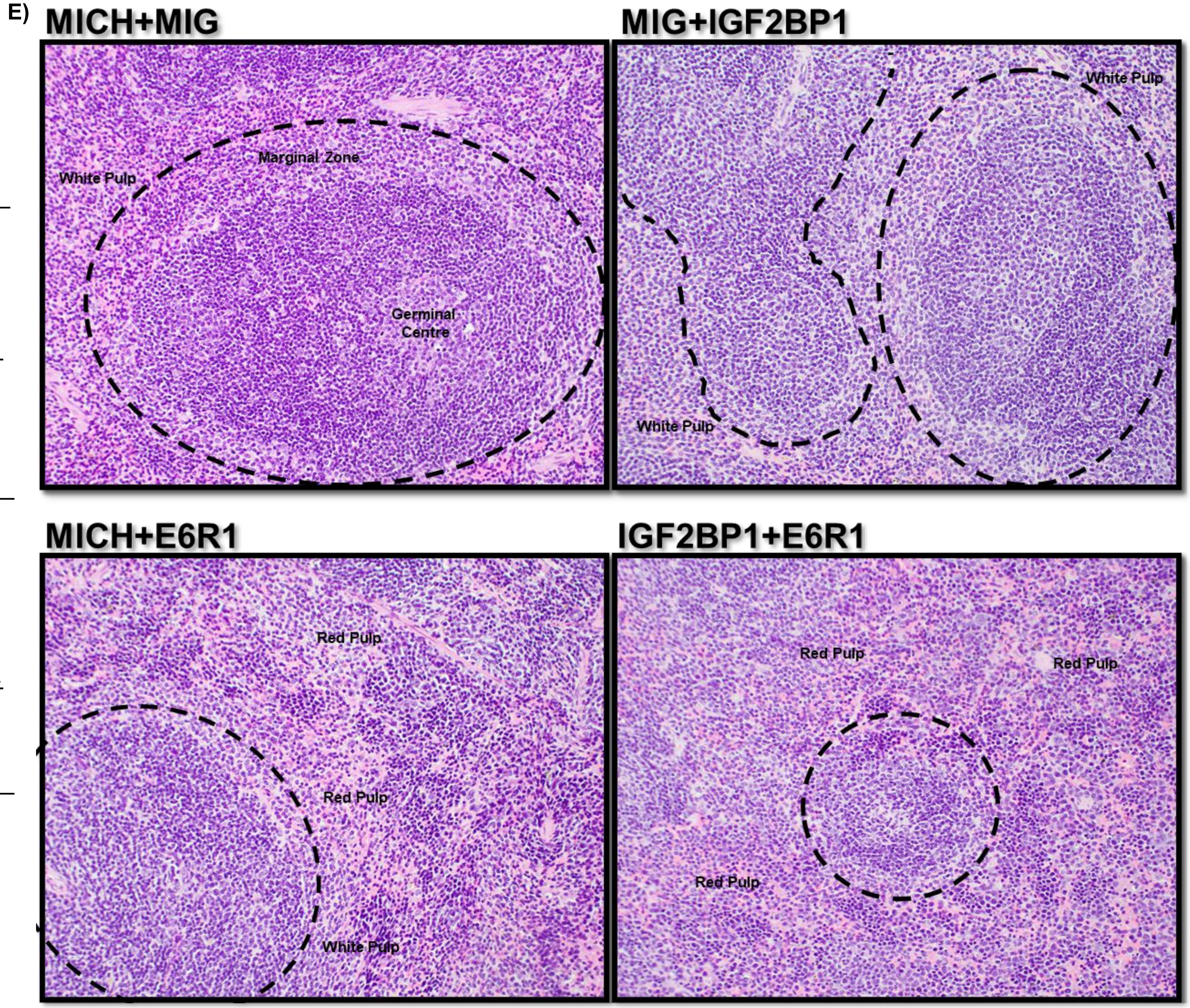

Figure S20: A) Spleen weights of mice belonging to different groups at week 16 B) Quantification of double positive ratio in the splenocytes and C) Lin- population by the ratio of GFP+ mCherry+ double positive cells D) Quantification of HSCs in the spleen (t-test; p *<0.05, ** <0.01, *** <0.005, **** <0.0001) E) Histological analysis of spleens of mice belonging to different groups; Controls show a prominent germinal center and marginal zone with clear white and red pulp distinction; Red pulp expansion, smaller germinal centers and loss of architecture is seen in IGF2BP1+ETV6::RUNX1 combination (200X)

SUPPLEMENTARY METHODS

Patient/Control sample collection and processing

Patient Inclusion Criteria: newly diagnosed B-ALL, age range from 0 to 18 years. Exclusion Criteria: Patients treated elsewhere initially or ALL developing secondary to another malignancy. The bone marrow sample collected was subjected to RBC Lysis Buffer for 30 min at 4° C under sterile conditions inside a laminar flow hood. Afterwards, the samples were centrifuged at 130 RCF for 10 mins at 4° C and washed with 1X Phosphate Buffer Saline (Gibco) followed by centrifugation. The cell pellet was resuspended in 1 mL of TRIzol (Thermo Fisher) followed by RNA isolation. The peripheral blood from healthy population was also drawn after proper consent and subjected to the same procedures as described for BM sample processing followed by RNA isolation.

CD19+ B cells were isolated from human PBMCs using CD19 MicroBeads from Miltenyi Biotech as per the manufacturer's protocol (1). Briefly 10 mL peripheral blood was drawn after consent of the control subjects and subjected to RBC lysis. The WBC pellet was incubated with anti-CD19 magnetic beads. Labelled cells were enriched by passing the cell suspension through an LS column (Miltenyi Biotech) under the influence of a magnetic field. The purity of enriched CD19+ B-cells was analyzed using flowcytometry before proceeding for further experiments.

Cell Proliferation, drug cytotoxicity and viability assays

ETV6::RUNX1 and *IGF2BP1* KO (Knockout) and NT (Non-Targeting Control) cells were seeded at 20000 cells/well in 96-well plates and cultured for 72 hours at 5% CO_2 and 37°C. MTS reagents were added according to the manufacturer's instructions (Promega CellTiter 96 AQueous Non-Radioactive Cell Proliferation Assay kit) and cells were incubated at 37°C, 5% CO2 for 4 hours before absorbance was measured at 490 nm

(Tecan, USA). Five technical replicates were used per sample per condition. Cytotoxicity and IC50 were measured using the MTS assay as above.

Cloning

The *IGF2BP1* CDS (from cDNA of Reh cell line) and *ETV6::RUNX1* fusion RNA were amplified using Hot-Start KOD DNA Polymerase (Merck) as per the manufacturer's protocol. *ETV6::RUNX1* was subcloned from pcDNA3.1- *ETV6::RUNX1*, a kind gift from Dr Anthony Ford at the Institute for Cancer Research (ICR), London.

The amplified fragments were gel extracted using Qiagen DNA Gel Extraction kit according to the manufacturer's protocol. The purified products were cloned in bicistronic, retroviral, murine overexpression vectors having GFP and mCherry as fluorescent markers. *ETV6::RUNX1* was cloned in MIG (MSCV-MCS-IRES-GFP) using BglII and EcoRI restriction sites and *IGF2BP1* was cloned in MICH (MSCV-MCS-IRES-mCherry) using BamHI and EcoRI restriction sites respectively. Plasmids were verified by Sanger sequencing and used for further experiments.

Western blotting

Cells were lysed in RIPA buffer supplemented with Halt Protease and Phosphatase Inhibitor Cocktail (Thermo Scientific). Equal amounts of protein lysate (as quantified by using bicinchoninic acid protein assay, BCA (Thermo Scientific)) were electrophoresed on a 5%–12% SDS-PAGE and electroblotted onto a nitrocellulose membrane. Antibodies used were IGF2BP1 rabbit polyclonal (RN077, MBL, Japan), IGF2BP3 rabbit polyclonal (RN009, MBL, Japan), NF-kB1 p105/50 (D7H5M) rabbit monoclonal (12540S, CST), IKKα (3G12) mouse monoclonal (11930S, CST), Akt (pan) (C67E7) rabbit monoclonal (4691T, CST), Phospho-Akt (Thr308) (D25E6) rabbit monoclonal (13038T, CST), MDM2 (DIV2Z) rabbit monoclonal (86934S, CST), GSK3β (27C10) rabbit monoclonal (9315, CST), Phospho-GSK-3β (Ser9) rabbit monoclonal (9336, CST), PDK1 rabbit monoclonal

(MA45-15797 ThermoFischer Scientific), Phospho-PDK1(Ser241) rabbit (3061, CST), GAPDH (D16H11) polyclonal rabbit monoclonal (5174, CST) and β-actin (catalogue AC15, Sigma-Aldrich) mouse monoclonal. Secondary HRP-conjugated antibodies from Santa Cruz Biotechnology Inc. & Cell Signaling Technology were used. SuperSignal West Pico kit (Pierce Biotechnology) and WESTAR ANTARES ECL (CYANAGEN (XLS142,0250) were used for enhanced chemiluminescence-based detection.

Bone Marrow transplant experiment

Retroviral vectors MICH/MICH-IGF2BP1 and MIG/MIG-ETV6::RUNX1 CDS were co-transfected with the packaging vector pCL-Eco in HEK-293T cells using Lipofectamine 3000 (Invitrogen). Supernatants containing retroviruses generated from HEK-293T cells were collected and filtered (0.45μM syringe filter) at 36 hours and 48 hours post-transfection and stored at 4^0C.

All mouse experimental procedures were conducted with the approval of the UCLA Chancellor's Animal Research Committee (ARC). BM was harvested and spin infected from 8-week-old 5-fluorouracil (5-FU) injected CD45.2+ donor C57BL/6J female mice as previously described (2). The harvested bone marrow cells were plated in 24 well plates in (10^6 cells per well) cytokine rich media and were subjected to spin infection at 30^0C and 2500g for 90 mins in the presence of 10 μg/mL polybrene for 3 consecutive days with each of the following retroviruses:

S.No.	Group	Day 1	Day 2	Day 3 (1:1 mixture)
1	Control	MIG	MICH	MIG+MICH
2	E6R1	MIG-E6R1	MICH	MIG-E6R1+MICH
3	IGF2BP1	MICH-IGF2BP1	MIG	MICH-IGF2BP1+MIG
4	IGF2BP1+E6R1	MICH-IGF2BP1	MIG-E6R1	MICH-IGF2BP1+MIG-E6R1

Eight-week-old CD45.1+ recipient B6.SJL-Ptprc-Pep3/BoyJ female mice were lethally irradiated (1050 Rads) and retro-orbitally injected with donor BM 16 hours after irradiation. Eight mice were used per group per experiment. Mice were bled at 4, 8, 12 and 16 weeks after BM injection for analysis of the peripheral blood. All mice were purchased from the Jackson Laboratory and housed under pathogen free conditions at UCLA.

Flow cytometry

The mice were euthanized at 16 weeks post-transplant. PB (peripheral blood), BM and spleen were collected. Single cell suspensions were lysed in red blood cell lysis buffer. Fluorochrome-conjugated antibodies were used for staining. The list of antibodies used is provided in Supplementary Table 4. Various surface markers were used to analyze the progenitor and mature populations in the bone marrow and spleen as described previously (3, 4).

For intracellular staining, after initial staining with surface marker antibodies and fixation with 1% paraformaldehyde (PFA), cells were incubated with antibodies against intracellular antigen Ki67 in 1% Triton containing MACS buffer. After 30 minutes of staining at 4°C, cells were washed twice with PBS and fixed with 1% PFA. Flow cytometry was performed at the UCLA JCCC and at the Broad Stem Cell Research Flow Core. Analysis was performed using FlowJo software.

Histopathology

Fixation and sectioning have been described previously (2). Analysis was performed by a board-certified hematopathologist (DSR).

RNA immunoprecipitation (RIP)

RIP was performed according the protocol standardized previously in our lab (5). Briefly, Reh cells were lysed in a cocktail of Non-Denaturing Lysis Buffer and Protease plus RNAse Inhibitors. 10% of the precleared lysate was stored as 'Input'. The precleared lysates were incubated overnight with Protein G Agarose beads along with antibody against human IGF2BP1 (MBL RN007P) or normal Rabbit IgG (CST 2729). Pelleted beads were washed thrice with PBS and resuspended in Non-Denaturing Buffer the next day. RNA was extracted using QIAGEN miRNeasy RNA isolation kit as per the manufacturer's protocol. A part of the RNA samples was subjected to reverse transcription and qPCR and the rest was sent for RNA sequencing (Input RNA and IGF2BP1 immunoprecipitated RNA).

RNA-Seq, RIP-Seq and Data Analysis

RNA was purified from both IGF2BP1-depleted or control Reh cells using the miRNEasy kit (Triplicates). For the RIP, three input and three IGF2BP1 immunoprecipitated samples were used for RNA isolation. Purified RNA was converted to libraries (1x50bp) and sequenced on Illumina HiSeq 3000 platform. All the sequencing were done in the Technology Centre for Genomics & Bioinformatics (TCGB) at UCLA. The data generated corresponded to approximately 30–50 million reads per sample. The STAR ultrafast universal aligner v2.7.3a (6) was used to generate the genome index and perform single-end alignments. Reads were aligned to a genome index that includes both the genome sequence (GRCh38 primary assembly) and the exon/intron structure of known gene models (Gencode genome annotation version 33). Alignment files were used to generate gene-level count summaries with STAR's built-in gene counter. Only protein-coding genes in the Gencode 33 annotation were considered (>97% of total counts for all samples). Independent filtering was applied as before (7): genes with less than one average count per sample, count outliers or low mappability were filtered out for

downstream analysis. Supplementary tables provide per-sample normalized data in units of Transcripts per Million (TPMs) after correcting for gene mappable length and per-sample sequencing depth. However, all downstream analysis including ordination, clustering and differential expression analyses was performed on count-based normalized and variance-stabilized data obtained with the DESeq2 (8) package unless otherwise noted.

Principal component analysis (PCA) of RNA-Seq and RIP-Seq samples was performed with the function prcomp in R (9) using variance-stabilized data as input. The first principal component (PC1) clearly discriminated between wild-type and *IGF2BP1* KO samples in RNA-Seq, and between IP and input samples in the IGF2BP1 RIP-Seq screen (more than 75% of total variance for both datasets). Therefore, for the Gene Set Enrichment Analysis (10) (GSEA) presented throughout the manuscript, gene ranks and gene sets were selected according to signed PC1 loadings. In addition, GSEA was also used to estimate enrichment on hallmark pathways from the Molecular Signature Database (MSigDB) (11, 12) and independent microarray experiments (5). Additional functional enrichment was performed with Metascape (13) and enrichment statistics presented as hypergeometric adjusted p-values. All plots were generated in R (9) and Matlab (MATLAB, version release 2020b, The MathWorks, Inc, RRID:SCR_001622).

For functional enrichment of RIP-Seq derived targets, we used the web-based version of SETEN9, a tool for systematic identification and comparison of processes, phenotypes and diseases associated with RNA-binding protein. Results from DESeq2 analysis were used as input. SETEN uses a permutation-based gene set enrichment method for analysis. The scores of common genes are compared with the scores of randomly picked genes using Mann Whitney U test, and the final p-value is computed by max (1 – # sign. tests / # total tests, 1 / # total tests). SETEN integrates functional and gene set enrichment

results using Fisher's method of p-value integration to assign a single p-value for each gene set. The output is presented in the form of bar graphs, tables and charts.

To identify IGF2BP1 targets which are overexpressed in *ETV6::RUNX1* positive B-ALL patient samples we used the following methodology. We reanalyzed the following datasets:

1. GSE65647 where *ETV6::RUNX1* positive patient samples (n=14) were compared with *E2A-PBX1* positive samples (n=15) and *MLL* translocated samples (n=15) by a microarray.
2. A dataset which compared *ETV6::RUNX1* positive patients' gene expression (n=4) with normal CD19 positive B-cells (n=2) (14)

Enriched pathways and genes specific to *ETV6::RUNX1* positive B-ALL were identified from these two datasets and intersected with our *IGF2BP1* KO RNA-Seq and IGF2BP1 RIP-Seq datasets. The final gene list included only the significantly enriched targets ($p<0.05$). Genes which showed significant higher differential fold change in the first two datasets and respective lower differential fold change in the *IGF2BP1* KO dataset along with a significant peak enrichment in the RIP dataset were selected for validation.

Luciferase Assay

IGF2BP1 CDS was cloned in a pHAGE6-dsRed-IRES-ZsGreen vector by replacing the dsRed fragment using NotI and BamHI restriction sites. The *ETV6::RUNX1* fusion junction of 660 bp was cloned downstream of firefly luciferase in the pMirGlo vector using NheI and XhoI restriction sites. All retro/lentiviral vectors were confirmed by Sanger sequencing before their use in the experiments.

293T cells were transfected with the pMirGlo, *ETV6::RUNX1* containing reporter vectors along with the pHAGE6-empty vector, pHAGE6-IGF2BP1 overexpression vector at a 1:10 ratio (50:500 ng) using Lipofectamine as described earlier (5). Cells were lysed after 48 hours, and luminescence was measured on a GloMax instrument (Promega). The ratio of firefly to Renilla luciferase activity was calculated for all samples. Luminescence was normalized to the empty vector controls.

NFκB Activation Assay

In order to study the cellular NFκB activity, we used a lentiviral vector with an NFκB responsive element upstream of a luciferase reporter. Replication incompetent lentiviruses of pHAGE-NFκB-TA-Luc-Ubc-dTomato were created using a 5-plasmid transfection procedure as previously described (15). Reh cells were transduced with these lentiviruses by spin infection as described previously (2).

Reh- NFκB-TA-Luc-Ubc-dTomato cells were incubated with different doses of BTYNB or DMSO (5 μM and 7.5 μM) for 48 hours. After 48 hours, cells were treated with 25 ng/ml TNFα (Tocris) for 3 hours. Firefly Luciferase activity was quantified using the DualGlo luciferase assay system and Glomax reader (Promega).

Supplementary Table 1. List of primers used in this study

Gene amplified	Primers used	Sequence (5’-3’)
IGF2BP1	IGF2BP1 F<br>IGF2BP1 R	5' TTA CTG GGG CTG CTC CCT AT 3'<br>5' TTC GGG TGG TGC AAT CTT GA 3'
ETV6::RUNX1	ETV6-A F<br>RUNX1-B R	5' TGC ACC CTC TGA TCC TGA AC 3'<br>5' AAC GCC TCG CTC ATC TTG C 3'
RNA Polymerase II	POL2A F<br>POL2A R	5' CAT CAA GAG AGT CCA GTT CGG 3'<br>5' CCC TCA GTC GTC TCT GGG TA 3'
NFKB2	NFKB2 F<br>NFKB2 R	5' AGA GGT GAA GGA AGA CAG TGC 3'<br>5' TTG AAA TAG GTG GGG ACG CTG 3'
NFAT5	NFAT5 F<br>NFAT5 R	5' CAG GCC AAC CAC AAA ACG AG 3'<br>5' AGC CAG TCA AGT TGT TCC CT 3'
IL6ST	IL6ST F<br>IL6ST R	5' TGC AGT TTG TGT GCT AAA GGA A 3'<br>5' CTG TAA AGG TGA CAC TGG ATG C 3'
GADD45A	GADD45A F<br>GADD45A R	5' GGG CCC GGA GAT AGA TGA CT 3'<br>5' TAC CCA AAC TAT GGC TGC ACA 3'
CDK6	CDK6 F<br>CDK6 R	5' GCT GAC CAG CAG TAC GAA TG 3'<br>5' CTT CAA CGC CAC GAA ACG G 3'
MDM2	MDM2 F<br>MDM2 R	5' AGA TGT TGG GCC CTT CGT GA 3'<br>5' CAA AGC CCT CTT CAG CTT GTG 3'
NGFR	NGFR F<br>NGFR R	5' TAC CAG GAT GAG ACG ACT GGG 3'<br>5' TTG GCC TCG TCG GAA TAC G 3'
CCND1	CCND1 F<br>CCND1 R	5' CCA ACC TCC TCA ACG ACC G 3'<br>5' CAT CCA GGT GGC GAC GAT CT 3'

ETV6<br>(RIP Primer)	ETV6 5' UTR F<br><br>ETV6 5' UTR R	5' GGG AGA GAT GCT GGA AGA AA 3'<br><br>5' GCT ACA CTG AGC AGG AGT CTC A 3'
LIMS2<br>(RIP Primer)	LIMS2 3' UTR F<br><br>LIMS2 3' UTR R	5' CAC CTC CAA TCC CTC ACC AC 3'<br><br>5' CCT GGA GGT GGG AAC AAG TC 3'
MYC<br>(RIP Primer)	MYC CDS F<br><br>MYC CDS R	5' CAT CAG CAC AAC TAC GCA GC 3'<br><br>5' TCG TTT CCG CAA CAA GTC CT 3'
ACTB<br>(RIP Primer)	ACTB 3' UTR F<br><br>ACTB 3' UTR R	5' ACC TAA CTT GCG CAG AAA ACA 3'<br><br>5' GCA ATC AAA GTC CTC GGC CA 3'
PPIA	PPIA F<br><br>PPIA R	5' CAC CGT GTT CTT CGA CAT TG 3'<br><br>5' TTC TGC TGT CTT TGG GAC CT 3'
Igf2bp1	mIGF2BP1 F<br><br>mIGF2BP1 R	5' GAG AAC TGT GAG CAA GTG AAC AC 3'<br><br>5' ATG TAG GAG ACC TTC AGG GCA T 3'
L32	mL32 F<br><br>mL32 R	5' AAG CGA AAC TGG CGG AAA C 3'<br><br>5' TAA CCG ATG TTG GGC ATC AG 3'
Gapdh	mGAPDH F<br><br>mGAPDH R	5' CGA CTT CAA CAG CAA CTC CCA CTC TTC C 3'<br><br>5' TGG GTG GTC CAG GGT TTC TTA CTC CTT 3'
FGFR1	FGFR F<br><br>FGFR R	5' CAG GGG AGG ATT CCG TCT TC 3'<br><br>5' CTG TGG GTG AGG GTT ACA GC 3'
HGPRT	HGPRT F<br><br>HGPRT R	5' CGT CTT GCT CGA GAT GTG ATG 3'<br>5' TGT AAT CCA GCA GGT CAG CAA A 3'

Supplementary Table 2. List of guide RNAs used in this study

Gene amplified	Primers used	Sequence 5’-3’
ETV6::RUNX1 sg1	ETV6::RUNX1 ex2 OLIGO 1	5’ CAC CGG GCG TCG AGG AAG CGT AAC T 3’
	ETV6::RUNX1 ex2 OLIGO 2	5’ AAA CAG TTA CGC TTC CTC GAC GCC C 3’
ETV6::RUNX1 sg2	ETV6::RUNX1 ex4 OLIGO 1	5' CAC CGA ATG GTG AAA AAA GAA TCC G 3'
	ETV6::RUNX1 ex4 OLIGO 2	5' AAA CCG GAT TCT TTT TTC ACC ATT C 3'
ETV6::RUNX1 sg3	ETV6::RUNX1 ex5 OLIGO 1	5' CAC CGT CCA TGG GAG ACA CTG ACA G 3'
	ETV6::RUNX1 ex5 OLIGO 2	5' AAA CCT GTC AGT GTC TCC CAT GGA C 3'
IGF2BP1 sg1	IGF2BP1 ex7 OLIGO 1	5’ CAC CGC AAG ATC ATC TTA CAA GCG G 3’
	IGF2BP1 ex7 OLIGO 2	5’ AAA CCC GCT TGT AAG ATG ATC TTG C 3’
IGF2BP1 sg2	IGF2BP1 ex9 OLIGO 1	5’ CAC CGG GCC ATC GAG AAT TGT TGC A 3’
	IGF2BP1 ex9 OLIGO 2	5’ AAA CTG CAA CAA TTC TCG ATG GCC C 3’
IGF2BP1 sg3	IGF2BP1 ex5 OLIGO 1	5’ CAC CGA ATG TCA CCT ATT CCA ACC 3’
	IGF2BP1 ex5 OLIGO 2	5’ AAA CGG TTG GAA TAG GTG ACA TTC 3’
NT 1	Control OLIGO 1	5’ ACC GGG CGA GGA GCT GTT CAC CG 3’
	Control OLIGO 2	5’ AAA CCG GTG AAC AGC TCC TCG CC 3’
NT 2	Control OLIGO 3	5’ ACC GCG AGG TAT TCG GCT CCG CG 3’
	Control OLIGO 4	5’ AAA CCG CGG AGC CGA ATA CCT CG 3’

Supplementary Table 3. Surface markers for hematopoietic progenitors

Full definition	Abbreviation	Marker Profile
Hematopoietic Stem Cell	HSC	Lin^-, c-Kit ^{high}, Sca-1^{high}, $CD150^+$
Lymphoid-primed Multipotent Progenitors	LMPP	Lin^-, c-Kit ^{high}, Sca-1^{high}, $Flt3^+$, IL-7Rα$^-$
Common Lymphoid Progenitor	CLP	Lin^-, c-Kit ^{low}, Sca-1^{low}, $Flt3^+$, IL-7Rα$^+$
Megakaryocyte/Erythroid Progenitor	MEP	Lin^-, c-Kit ^{high}, Sca-1^-, $CD34^{low}$, $CD16/CD32^{low}$
Common Myeloid Progenitor	CMP	Lin^-, c-Kit ^{high}, Sca-1^-, $CD34^{high}$, $CD16/CD32^{low}$
Granulocyte/Monocyte Progenitor	GMP	Lin^-, c-Kit ^{high}, Sca-1^-, $CD34^{high}$, $CD16/32^{high}$

Supplementary Table 4. Hardy fractions Surface markers

Fraction	Subset	Marker Profile
A	Pre-Pro-B	$B220^+$, IgM^-, $CD43^+$, $CD24^-$, $Ly51^-$
B	Pro-B	$B220^+$, IgM^-, $CD43^+$, $CD24^+$, $Ly51^-$
C	Pro-B/ Large Pre-B	$B220^+$, IgM^-, $CD43^+$, $CD24^+$, $Ly51^+$
D	Small Pre-B	$B220^+$, $CD43^-$, IgM^-
E	Immature B	$B220^+$, $CD43^-$, IgM^+

Supplementary Table 5. List of antibodies used in flowcytometry

Lineage	HSC CLP	Hardy Fractions
APC CD3e	CD127-PE-Cy7	IgM-BV711
PerCP-Cy5.5 B220	Ly6A/Sca1-PerCP-Cy5.5	B220-PerCP Cy5.5
PE-Cy7 CD11b	CD117 (c-kit)-APC-Cy7	CD24-PE-Cy7
APC-Cy7 CD45.1	CD150-BV711	CD43-APC
**CMP**	CD135 (Flt3)-APC	Ly51-Biotin
CD16/32-PE-Cy7	Biotin-CD8	Streptavidin-eFluor-450
Ly6A/Sca1-PerCP-Cy5.5	Biotin-CD4	**Spleen Fractions**
CD117 (c-kit)-APC-Cy7	Biotin-B220	IgM-BV711
CD34-Alexa Fluor 700	Biotin-TCRβ	CD93-APC
CD127-APC	Biotin-TCRγδ	CD23-PE-Cy7
Biotin-CD8	Biotin-NK1.1	CD21-APC-Cy7
Biotin-CD4	Biotin-IgM	B220-PerCP-Cy5.5
Biotin-B220	Biotin-Gr1	
Biotin-TCRβ	Biotin-Ter119	
Biotin-TCRγδ	Streptavidin-eFluor-450	
Biotin-NK1.1		
Biotin-IgM		
Biotin-Gr1		
Biotin-Ter119		
Alexa Fluor 532 Streptavidin		

References

1. *Moore, DK, Motaung, B, du Plessis, N, Shabangu, AN, Loxton, AG, Consortium, S-I. Isolation of B-cells using Miltenyi MACS bead isolation kits. PLOS ONE. 2019; 14:e0213832.*
2. *O'Connell, RM, Rao, D.S., Chaudhuri, A.A., Boldin, M.P., Taganov, K.D., Nicoll, J., Paquette, R.L., Baltimore, D. Sustained expression of microRNA-155 in hematopoietic stem cells causes a myeloproliferative disorder. Journal of Experimental Medicine. 2008; 205:585–594..*
3. *Contreras, JR, Palanichamy, JK, Tran, TM, Fernando, TR, Rodriguez-Malave, NI, Goswami, N, et al. MicroRNA-146a modulates B-cell oncogenesis by regulating Egr1. Oncotarget. 2015; 6:11023-37.*
4. *Fernando, TR, Contreras, JR, Zampini, M, Rodriguez-Malave, NI, Alberti, MO, Anguiano, J, et al. The lncRNA CASC15 regulates SOX4 expression in RUNX1-rearranged acute leukemia. Mol Cancer. 2017; 16:126.*
5. *Palanichamy, JK, Tran, TM, Howard, JM, Contreras, JR, Fernando, TR, Sterne-Weiler, T, et al. RNA-binding protein IGF2BP3 targeting of oncogenic transcripts promotes hematopoietic progenitor proliferation. J Clin Invest. 2016; 126:1495-511.*
6. *Dobin, A, Davis, CA, Schlesinger, F, Drenkow, J, Zaleski, C, Jha, S, et al. STAR: ultrafast universal RNA-seq aligner. Bioinformatics. 2013; 29:15-21.*
7. *Casero, D, Sandoval, S, Seet, CS, Scholes, J, Zhu, Y, Ha, VL, et al. Long non-coding RNA profiling of human lymphoid progenitor cells reveals transcriptional divergence of B cell and T cell lineages. Nat Immunol. 2015; 16:1282-91.*
8. *Love, MI, Huber, W, Anders, S. Moderated estimation of fold change and dispersion for RNA-seq data with DESeq2. Genome Biol. 2014; 15:550.*
9. *R Core Team. R: A Language and Environment for Statistical Computing. R Foundation for Statistical Computing. 2019.*
10. *Subramanian, A, Tamayo, P, Mootha, VK, Mukherjee, S, Ebert, BL, Gillette, MA, et al. Gene set enrichment analysis: a knowledge-based approach for interpreting genome-wide expression profiles. Proc Natl Acad Sci U S A. 2005; 102:15545-50.*

11. *Liberzon, A, Subramanian, A, Pinchback, R, Thorvaldsdóttir, H, Tamayo, P, Mesirov, JP. Molecular signatures database (MSigDB) 3.0. Bioinformatics. 2011; 27:1739-40.*

12. *Liberzon, A, Birger, C, Thorvaldsdóttir, H, Ghandi, M, Mesirov, JP, Tamayo, P. The Molecular Signatures Database (MSigDB) hallmark gene set collection. Cell Syst. 2015; 1:417-25.*

13. *Zhou, Y, Zhou, B, Pache, L, Chang, M, Khodabakhshi, AH, Tanaseichuk, O, et al. Metascape provides a biologist-oriented resource for the analysis of systems-level datasets. Nat Commun. 2019; 10:1523.*

14. *Linka, Y, Ginzel, S, Kruger, M, Novosel, A, Gombert, M, Kremmer, E, et al. The impact of TEL-AML1 (ETV6-RUNX1) expression in precursor B cells and implications for leukaemia using three different genome-wide screening methods. Blood Cancer J. 2013; 3:e151.*

15. *Wilson, AA, Kwok, LW, Hovav, A-H, Ohle, SJ, Little, FF, Fine, A, et al. Sustained expression of alpha1-antitrypsin after transplantation of manipulated hematopoietic stem cells. Am J Respir Cell Mol Biol. 2008; 39:133-41*.
